# iHEART trial: study protocol for a German multicentre randomised controlled trial on the feasibility and acceptance of an internet-based preoperative intervention to optimise patient expectations and improve outcomes after heart surgery

**DOI:** 10.1136/bmjopen-2024-092482

**Published:** 2025-09-17

**Authors:** Viviane Noelani Compère, Sümeyye Balci, Christine Heinz, Carmen Schade-Brittinger, Ardawan J Rastan, Miriam Salzmann-Djufri, Bernd Niemann, Andreas Boening, Yeong-Hoon Choi, Anna-Carlotta Zarski, Johannes Laferton, Frank Euteneuer, Winfried Rief, Stefan Salzmann

**Affiliations:** 1 Division of Clinical Psychology and Psychotherapy, University of Marburg, Marburg, Germany; 2 Coordinating Center for Clinical Trials (KKS), University of Marburg, Marburg, Germany; 3 Department for Cardiovascular Surgery, Heart Center, University Hospital of Giessen and Marburg, Marburg, Germany; 4 Department for Cardiovascular Surgery, University Hospital Giessen, Giessen, Germany; 5 Department of Cardiac Surgery, Department of Cardiology & Department for Heart Surgery, Kerckhoff Clinic Heart Centrum, Bad Nauheim, Germany; 6 Department of Clinical Psychology, Division of eHealth in Clinical Psychology, University of Marburg, Marburg, Germany; 7 Institute for Mental Health and Behavioral Medicine & Department of Medicine, HMU Health and Medical University Potsdam GmbH, Potsdam, Germany; 8 Department of Psychology, Clinical Psychology and Psychotherapy, MSB Medical School Berlin GmbH, Berlin, Germany; 9 Medical Psychology, HMU Health and Medical University Erfurt GmbH, Erfurt, Germany

**Keywords:** Coronary heart disease, Cardiovascular Disease, Clinical Trial, eHealth, Feasibility Studies, Cardiac surgery

## Abstract

**Introduction:**

Treatment expectations are a key mechanism of placebo effects in clinical trials. In a previous study (PSY-HEART-I), preoperative expectation optimisation improved quality of life 6 months postcardiac surgery. However, barriers such as travel distance, staffing shortages and COVID-19 limited participation. This study evaluates the feasibility and acceptability of iEXPECT, a brief internet-based intervention designed to optimise expectations before heart surgery.

**Methods and analysis:**

In this three-arm, multicentre randomised controlled trial, 160 patients undergoing elective coronary artery bypass graft surgery are randomised to: (a) standard of care (SOC); (b) SOC plus iEXPECT with phone-based guidance (iEXPECT enhanced) or (c) SOC plus iEXPECT with email-based guidance (iEXPECT limited). The intervention includes four 20 min online modules addressing surgical benefits, side effects and coping strategies. Modules are accompanied by personalised guidance provided through feedback on each module via email or telephone (three before surgery, three booster sessions at 6, 12 and 18 weeks postsurgery). Assessments occur at baseline (5–21 days before surgery), preoperatively (day before surgery), 7 days postsurgery and 6 months later. Primary feasibility outcomes include recruitment (≥1 participant/week/centre), retention (≥49% completing 6-month follow-up including biomarkers) and engagement (≥75% completing ≥1 presurgery module). Acceptability is measured by self-reported enjoyment, usefulness and impact, with acceptance defined as mean scores >3.4 (5-point Likert scale) and CSQ-I ratings. Secondary outcomes include psychological measures, inflammatory markers and heart rate variability.

**Ethics and dissemination:**

Ethical approval was granted by the Ethics Committees of Philipps University Marburg (AZ 229/23 BO) and the University of Giessen (AZ 186/23). All participants provide written informed consent. Results will be shared via publications, conferences and public outreach with relevant consumer advocacy groups.

**Trial registration number:**

DRKS00033284.

**Strengths and limitations of this study**

- Involving three diverse clinical centres enhances generalisability and feasibility assessment across different hospital settings.
- Use of stratified, permuted block randomisation via REDCap, a secure platform for managing online databases and surveys, ensures allocation concealment and minimises selection bias.
- Medical staff (caregiver, treating physician) remain blinded to group assignment, reducing the risk of biased treatment effects.
- Although previous patient feedback informed intervention development, there was no structured patient involvement in designing the current trial.
- Digital intervention format excludes patients without internet access or sufficient digital literacy, potentially limiting inclusivity.

## Introduction

 One of the treatment options for patients with coronary heart disease (CHD) is coronary artery bypass grafting (CABG), where a surgically created blood vessel bridge aims to restore blood flow.^1^ While many individuals typically experience improvements in their overall health after heart surgery, not everyone has a smooth recovery. Some patients encounter challenges, such as diminished vitality, limited physical functioning, mediocre general health and bodily pain.^2^ For a substantial number of individuals, their health and quality of life may not show the expected improvement and may possibly deteriorate even further, despite the surgery being successful from a medical perspective.^3 4^ Preoperative expectations and illness beliefs play a crucial role in postoperative recovery and predict outcomes following heart surgery.^5^ These expectations, as one crucial mechanism of the placebo effect in clinical studies, are integral to medical procedures and contribute significantly to their success.^6 7^ This phenomenon extends beyond medication to surgeries and other medical procedures, underlining the pervasive influence of patient expectations.^8^,^10^

Enhancing patients’ expectations before surgery may help optimise postoperative outcomes. For this reason, the expectation-optimising intervention ‘EXPECT’ was developed as a preoperative psychological intervention to enhance patients’ benefit expectations, side-effect expectations and personal control/coping expectations (for a detailed intervention manual see^11 12^). The ‘EXPECT’ intervention comprised two face-to-face sessions and two telephone calls before the operation, complemented by a postoperative follow-up phone call.^12^ The pilot study (PSY-HEART-I trial) demonstrated that optimising preoperative expectations through the ‘EXPECT’ intervention had lasting positive effects on health outcomes, including reduced disease-related disability, improved quality of life, increased physical activity and shorter hospitalisation duration following bypass surgery.^13 14^ In the ongoing multicentric follow-up project (PSY-HEART-II trial), the effectiveness of the ‘EXPECT’ intervention is being explored on a broader scale at ten different study centres.^15^ Despite substantial demand, many patients faced barriers preventing their participation, such as long travel distances, scheduling issues and time constraints. The COVID-19 pandemic further complicated these efforts at surgical sites, underscoring the need for new strategies to improve preoperative preparation and ensure broader access to the intervention that can optimise treatment outcomes.

An internet-based adaptation of the ‘EXPECT’ intervention could offer significant benefits due to its scalability. By requiring fewer in-hospital resources and allowing patients to prepare from home, it can overcome geographical and time-related barriers, making optimal preoperative care accessible to a wider patient population.^16^ For a range of different mental disorders, therapist-guided internet-based cognitive-behavioural therapy has proven more effective than waitlist control conditions and has shown similar effectiveness to face-to-face interventions.^17^ Despite this success, no studies have examined the effects of an internet-based preoperative psychological intervention before heart surgery.

In a previous step, the traditional face-to-face ‘EXPECT’ intervention for expectation optimisation was adapted into an online format (iEXPECT). The present study—the iHEART trial—aims to test the feasibility of the study design and the acceptability of the online, preoperative, guided psychological iEXPECT intervention. Specifically, the primary objectives are to evaluate (1) the feasibility of implementing the study design across multiple sites, similar to the PSY-HEART-II trial, (2) whether patients are able and willing to engage with the intervention as intended and (3) the acceptability of the iEXPECT intervention from the patients’ perspective. Given the uncertainty around the optimal level of guidance for online interventions,^18 19^ this study will also explore two variations of the online intervention, each offering different levels of therapist support (enhanced vs limited interaction). Secondary outcomes include psychological and physiological measures that will be analysed descriptively per group. Biomarkers will be collected to assess the feasibility of obtaining inflammatory markers and heart rate variability (HRV).

This feasibility trial also serves as a precursor to a larger future study (Phase III trial) aimed at evaluating the efficacy of the iEXPECT intervention. If successful, the iHEART trial could counteract the previous barriers and could thus improve access to better healthcare for patients undergoing heart surgeries.

## Materials and analysis

### Registration and funding

The trial has been preregistered at the German Clinical Trials Register (https://www.drks.de/DRKS00033284). It is funded by the German Research Foundation (DFG) (SA 4505/3–1 PI Dr. Salzmann). For detailed information, see the online supplemental table S1, which contains all items of the WHO Trial Registration Data Set.

### Study design

**Figure 1:**
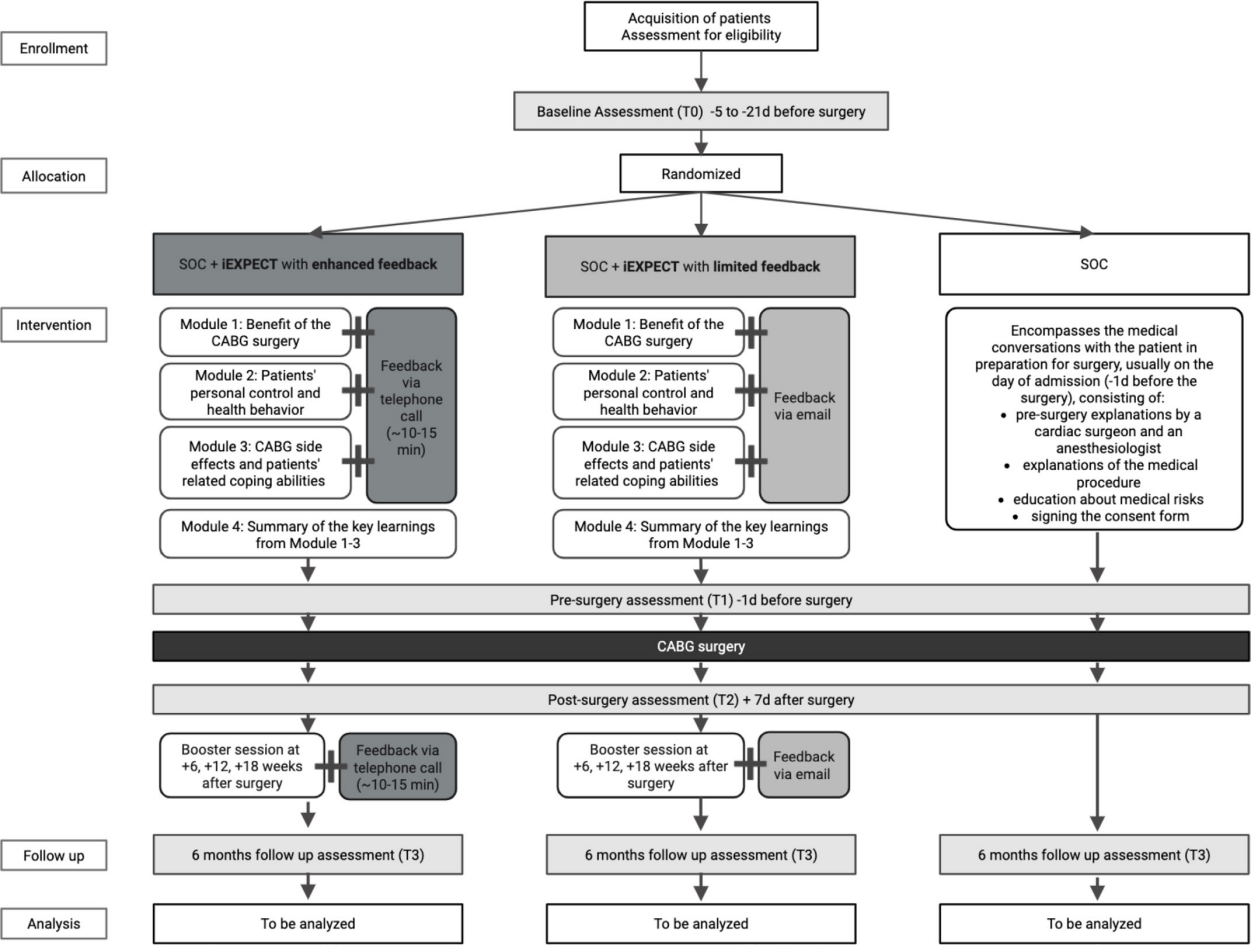
Study flow chart of the iHEART Study. Expectation manipulation online intervention (iEXPECT), standard of care (SOC), coronary artery bypass graft surgery (CABG), days (**d**). Assessments take place 5–21 days before the surgery (baseline, (**T0**), preoperatively on the day of hospital admission after the psychological intervention (**T1**), 7 days after surgery (**T2**) and follow-up, 6 months after surgery (**T3**). The iEXPECT interventions include four modules (20 min), of which the first three are guided, before the surgery, and three guided booster sessions 6, 12 and 18 weeks after the surgery.

The iHEART trial is a three-arm, multicentre, randomised controlled trial that examines the feasibility and acceptance of a guided preoperative internet-based intervention aiming to optimise treatment outcome expectations (iEXPECT) in addition to standard of care (SOC). The content, derived from the face-to-face ‘EXPECT’ intervention,^11 15 20^ aims to improve individual expectations, enhancing recovery for cardiac surgery patients through placebo effects.^12 14 20^ Adapted to an online format, the intervention covers three main components to promote personalised, positive, realistic expectations and correct dysfunctional beliefs: (1) benefit expectations of surgery, (2) personal control strategies (eg, health behaviour, lifestyle) and (3) coping with negative postoperative effects (see table 1). These aspects of the intervention are expected to influence psychological factors and yield improvements such as reducing anxiety levels or depressive symptoms. As part of the intervention, guidance is provided through personalised feedback via phone calls (iEXPECT enhanced) or via email (iEXPECT limited). Participants will be recruited and data collected at the three participating centres: the university hospital of Marburg, the university hospital of Gießen and the medical centre Kerckhoff-clinic, Bad Nauheim. Figure 1 shows the study flow chart.

**Table 1:** Module overview of the preoperative psychological iEXPECT online intervention

Module names	Content
Benefits of the CABG surgery	Welcome and introduction to the study platform (minddistrict.com). Psychoeducation on CHD and disease model. Benefits of the CABG surgery. Developing a personalised step-by-step recovery action plan (eg, go for a walk: in the first 6 weeks after surgery, lawnmowing: 6 weeks after surgery, heavy gardening: 3 months after surgery). Imagination exercise ‘6 months after a successful surgery—the best possible self’.
Personal control strategies (eg, health behaviour, lifestyle choices)	Information on risk factors and health behaviours. Individualised health behaviour plan. Emphasises personalised outcome expectations and self-efficacy. Imagination exercise ‘6 months after a successful surgery—the best possible self’.
Negative postoperative side effects and coping strategies	Temporary side effects like incision pain and waking in the ICU are discussed to reassure patients. Expectations for treatment outcomes and timelines are clearly explained. Developing personalised coping expectations. Imagination exercise ‘6 months after a successful surgery—the best possible self’.
Summary	Summary of the key content from modules 1–3. Imagination exercise ‘6 months after a successful surgery—the best possible self’.
Booster (1–3)	Key topics from the previous modules (eg, health behaviour maintenance, risk factors and personal control strategies). Gather feedback on complications or successful experiences. Imagination exercise ‘6 months after a successful surgery—the best possible self’.

Following modules (1–3) completion, patients receive personalised guidance via telephone call (iEXPECT enhanced) or via email (iEXPECT limited) on the content from the iHEART study team. All modules (at least 1–3) should be completed by no later than 1 day before the heart surgery.

CABG, coronary artery bypass graft; CHD, coronary heart disease; iEXPECT, expectation manipulation online intervention.

### Recruitment and enrolment

Recruitment was planned to start in April 2024, and the anticipated completion of recruitment (i.e., when the last patient is expected to be enrolled in the trial) is at the end of December 2025. The first patient was successfully recruited on 8 November 2024. To create a well-defined and homogeneous study population, and enhance internal validity, while ensuring participant safety, the following inclusion and exclusion criteria were applied:

#### Inclusion criteria

- Minimum age is 18 years.
- Patients undergoing their first elective aortocoronary bypass surgery (CABG, without valve) with or without the use of a heart-lung machine (extracorporeal circulation).
- Median sternotomy.
- Sufficient knowledge of the German language.
- Adequate cognitive fitness to provide consent for study participation.
- Valid email address.
- Regular access to a computer with an internet connection.
- Participants can provide informed consent at least 5–21 days before the surgery.

#### Exclusion criteria

- Emergency surgery.
- Minimally invasive surgical procedure.
- Presence of another (non-cardiac) life-threatening condition.
- Comorbid medical/psychiatric condition causing at least a similar impairment compared with coronary and valvular heart disease.
- Participation in other interventional/experimental research programmes.

### Procedure

The medical team at the Departments of Cardiac and Thoracic Vascular Surgery informs patients about the study as soon as their surgery is scheduled and they are confirmed to meet the key inclusion criteria—ideally between 21 and 5 days before their surgery. In addition to a brief phone call providing initial information, patients receive a brochure enclosed with their surgery confirmation letter. The brochure outlines the study’s objectives, procedures, duration and includes a link and QR code to the study website. On the website, patients complete anonymous screening questions to determine eligibility (eg, type of surgery, timing and whether it is their first heart surgery). Eligible patients are redirected to the detailed study information and informed consent page. Ineligible patients receive a message informing them they do not qualify for the study and continue to receive only the SOC without being provided any additional study-specific materials or guidance. All screening data—regardless of eligibility—are securely stored in the study database, with personal identifiers removed or coded in accordance with clinical data confidentiality standards.

#### Informed consent

On the informed consent page, patients receive comprehensive information about the study and participation conditions. They can express interest and request more information by providing contact details before giving written consent by ticking a box. Once initial consent is given, a study therapist contacts them by telephone to conduct a more detailed screening; during this call, patients are again informed about the study’s potential risks, have the opportunity to ask additional questions before giving full informed consent and being formally enrolled. During the phone call, the voluntary nature of participation and the option to withdraw at any time are emphasised. The risks of blood sampling are reiterated, and any remaining questions are clarified. As part of routine clinical care, patients are required to provide blood samples prior to surgery; participation in the optional research blood sampling component is voluntary, and patients may still enrol in the study even if they decline to provide study-specific specimens. If patients consent, the presence of relevant mental disorders is assessed using the International Diagnosis Checklists for ICD-10 and the Structured Clinical Interview for DSM-IV^21^ and any incidental findings are communicated to the study team. Patients may choose to be informed of suspected diagnoses, although the study does not expedite psychotherapy access; instead, general guidance on seeking care is provided, without a formal referral pathway. In urgent cases (eg, critical PHQ-9 scores), patients are contacted directly, if possible, in collaboration with the clinic, to ensure immediate support. Medical consent for heart surgery follows the clinic’s protocol.

### Randomisation

After baseline assessment, patients are randomised in a 2:2:1 ratio to the treatment groups (iEXPECT enhanced: iEXPECT limited: SOC) using permuted block randomisation, stratified by baseline disease-related impairment (Pain Disability Index (PDI), <23 or ≥23).^22^

Randomisation is conducted online via REDCap (https://www.project-redcap.org/), a secure web platform for managing online databases and surveys, on the servers of the Coordinating Centre for Clinical Trials (KKS), Philipps-University Marburg. A biometrician from the KKS, who had no involvement in trial analysis or data collection, developed the randomisation script using RStudio V.2022.12.0, and the KKS Data Management Department used this code to generate the final randomisation list. It is not planned to use back-up envelopes. Study therapists log into REDCap, verify that all relevant data (eg, informed consent, inclusion/exclusion criteria, baseline PDI value) are available for each patient, and initiate automated randomisation. Following randomisation, study therapists will grant participants access to the respective online intervention. The experimental groups can immediately begin the intervention. Participants are not explicitly instructed to conceal their group allocation from the treating medical team. However, the medical staff will not receive any information about the group a patient is allocated to and, therefore, be blind regarding group assignments.

### iEXPECT intervention

Participants in the intervention groups receive an email invitation with their patient IDs to access the online study platform (https://www.minddistrict.com/de-de) and instructions for use. They can progress through the intervention modules and contact the study team with questions.

**Figure 2:**
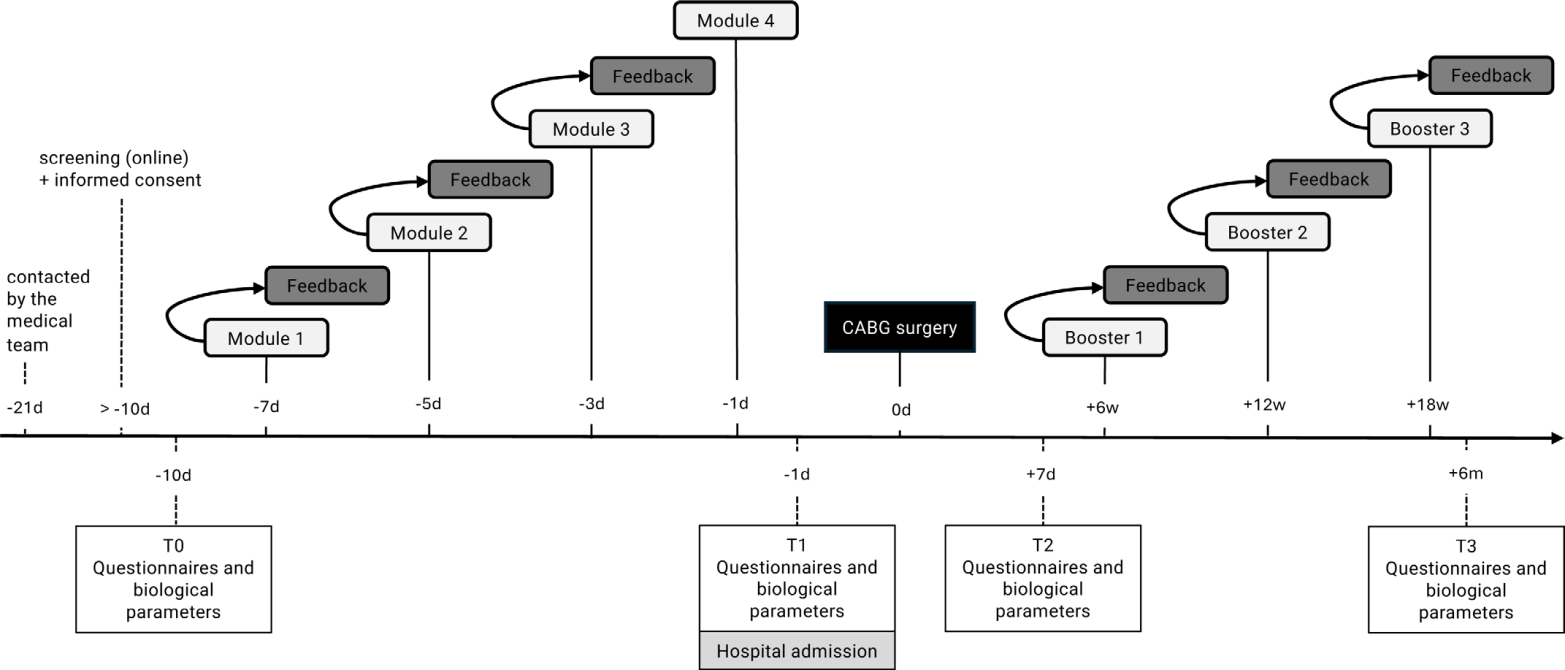
Intervention schedule for participants receiving the iEXPECT intervention. Days (**d**); weeks (**w**); months (**m**). Guidance consists of feedback on the previous module and is provided through email (iEXPECT limited) or a short phone call (~10-15 min; iEXPECT enhanced), within 1–2 working days after finishing each module. Modules 1–4 enforce positive and realistic expectations while addressing and correcting dysfunctional expectations and illness beliefs. Boosters recap key topics from the previous modules to sustain personal control expectations postoperatively. CABG, coronary artery bypass graft; iEXPECT, expectation manipulation online intervention.

The internet-based iEXPECT intervention with four modules before, and three guided booster sessions after surgery can be done from home in the participants’ own time (for an overview, please see the intervention schedule in figure 2). Participants receive semistructured guidance via email or phone (~10-15 min) within 1–2 working days after each module, reinforcing strategies and addressing challenges. Noteworthy, patients in the iEXPECT enhanced group will receive more tailored guidance as the personal contact via telephone allows study therapists to directly interact with the patients to ensure patients’ complete understanding. Furthermore, a second placebo mechanism (besides expectations) is used: positive interaction between patient and therapist.^23^

In the iHEART trial, the number of booster sessions is increased based on PSY-HEART-I findings, which showed that the ‘EXPECT’ interventions raised preoperative personal control expectations but patients struggled to maintain them postoperatively.^24^ The iEXPECT modules incorporate persuasive design elements^25 26^ to improve adherence. Examples of these elements include primary task support, such as exercises and homework; dialogue support, including calendars for upcoming modules and reminder emails or phone calls, depending on the experimental group a patient is in; and system credibility support, demonstrated through the inclusion of information sources and the logos of universities and clinics.

## Outcomes

### Primary outcomes

The primary outcomes of the iHEART trial are focused on evaluating the feasibility and acceptance of the iEXPECT intervention and the study design.

#### Feasibility

Feasibility is defined across two dimensions, namely (1) feasibility of the study design, referring to the successful recruitment and retention of participants across multiple study sites (outcome criterion 1) and (2) feasibility of intervention implementation, referring to the patients’ engagement with the iEXPECT modules prior to surgery (outcome criterion 2). These outcomes reflect whether the trial can be operationalised as intended and whether participants are willing and able to engage with the digital intervention components.

#### Acceptance

Acceptance is defined as participants’ subjective evaluation of the iEXPECT intervention (outcome criterion 3). This includes perceived enjoyableness, usefulness and the intervention’s relevance to their daily social functioning. Ratings are gathered through self-report at the end of each module and via a standardised satisfaction questionnaire (CSQ-I)^27^ after intervention completion (T1) and at 6-month follow-up (T3). Together, these outcomes will inform the practicality and perceived value of the intervention from the patient’s perspective and provide critical insight for the planning of a future full-scale efficacy trial.

### Secondary outcomes

Secondary outcomes include multimodal assessments (ie, self-ratings, expert ratings, clinical facts, biological measures) that were selected to capture a comprehensive picture of psychological, behavioural and clinical processes that may be affected by the intervention and are relevant for evaluating its broader impact and future Phase III trial feasibility. These include measures of preoperative anxiety and need for information (APAIS, Amsterdam preoperative anxiety and information scale),^28 29^ general anxiety and depression (PHQ9, patient health questionnaire; and GAD-7, generalized anxiety disorder scale),^30 31^ and treatment expectations (TEX-Q, treatment expectation questionnaire; and GEEE, generic rating scale for previous treatment experiences),^32 33^ all of which are known to influence surgical outcomes and psychological readiness. We also included constructs such as attitudes towards internet-based interventions (APOI, attitudes towards psychological online interventions),^34^ subjective illness beliefs (B-IPQ, brief-illness-perception questionnaire),^35^ social support (ESSI-D, enriched social support inventory-deutsch)^36^ and trait optimism (LOT-R, revised life orientation test),^37^ which may moderate treatment engagement and response. Furthermore, indicators of digital health literacy (GR-eHEALS, revised German eHealth literacy scale),^38^ physical activity (IPAQ, international physical activity questionnaire),^39^ perceived stress (PSS, perceived stress scale),^40 41^ illness-related disability caused by the current medical condition (PDI, pain disability index adapted version),^22^ pain (VAS, visual analogue scale, 0–10), quality of life (SF-12, short-form health survey)^42^ and treatment satisfaction (ie, rating of fulfilment/violation of pretreatment expectations at the end of the trial) may provide a multidimensional view of patient functioning across time. To explore potential adverse effects and unmet needs, we assess negative side effects (NEQ, negative effects questionnaire)^43^ and prior pretreatment experiences. The following clinical parameters are also included to explore associations between psychological factors and physical recovery: rehospitalisation; length of stay; length of time at intensive care unit; planned and realised surgery; EURO-Score II, European system for cardiac operative risk evaluation^44^; CSS score, Canadian Cardiovascular Society^45^; blood pressure; LVEF, left ventricular ejection fraction; New York heart association (NYHA) score; complications; inflammatory processes (C reactive protein (CRP), IL-6, Interleukin-6, IL-8, Interleukin-8); HRV, heart-rate-variability (ie, triangular index).

### Assessment

Assessments take place 5–21 days before the surgery (baseline, T0), preoperatively on the day of hospital admission after the psychological intervention (T1), 7 days after surgery (T2) and 6 months after surgery (T3) (for an overview, see figure 2). Psychological data will be collected through questionnaires (eg, regarding disease-related impairment, quality of life, depression and anxiety) and takes place online via the secure web-based survey platform UniPark (https://www.unipark.com/). At each assessment point (T0–T3), completion of the psychological questionnaires will require approximately 30 min. Participants are encouraged to complete them online via UniPark, although paper-based versions may be provided by clinical staff in cases of technical difficulties, and retention is supported through personalised email invitations, reminder emails and phone calls. These strategies have been shown to enhance compliance and reduce attrition,^46^ helping to ensure complete data collection across all time points. Patients will be asked to complete the questionnaires and to contact study therapists if they have any questions. For a detailed overview, see the online supplemental table S2, which shows the applied questionnaires, biological parameters and case report forms (CRFs). Furthermore, semistructured interviews with a small subset of CABG patients (up to N=12) will be conducted on a voluntary basis after surgery to retrospectively explore preparation needs and qualitatively assess the intervention. Interviews (30–45 min) will be audio-recorded, transcribed and analysed using qualitative content analysis.^47^ All data will be anonymised.

### Biological markers

Proinflammatory markers like CRP and interleukin-6 (IL-6) can induce depressive-like symptoms and alter neurophysiological processes.^48^ In PSY-HEART-I, the ‘EXPECT’ intervention reduced postoperative inflammation (interleukin-8) compared with standard treatment, with baseline inflammation moderating long-term outcomes.^49 50^ Biological factors are vital in understanding cardiovascular outcomes, especially since atherosclerosis, a common cause of CHD, raises inflammatory markers.^51 52^ The effectiveness of internet-based interventions in improving inflammation status remains unclear, warranting further research. HRV predicts heart surgery complications.^53^ This feasibility study will collect blood samples and HRV data at four timepoints: baseline (T0), postintervention (T1), postsurgery (T2) and 6 months postsurgery (T3) (see online supplemental table S2). To enhance feasibility, postoperative collection of biomarkers will be scheduled for postoperative day 7 or at the time of hospital discharge, whichever occurs first, to avoid requiring patients to return for bloodwork and HRV assessment after discharge.

#### Collection procedure

Blood samples will be taken outside of fasting periods to examine plasma levels of inflammatory markers (CRP, IL-6) at each assessment point (T0–T3). Patients will be either sitting or lying down to minimise the impact of movement or physical activity on the results. Prior to blood collection, patients will rest for 30 min. Once collected, blood will be placed into polyethylene tubes with EDTA as an anticoagulant (S-Monovette; Sarstedt, Nümbrecht, Germany), then promptly transported on ice to the laboratory. There, samples will be centrifuged at 2000 g for 10 min in a refrigerated centrifuge (4°C). Plasma aliquots will be stored at −80°C until analysis. Blood samples will be obtained by the study team in Marburg and sent to Essen for analysis by Prof. Schedlowski and his team at the Institute of Medical Psychology and Behavioral Immunobiology at the University Clinic Essen. Variability coefficients will be calculated for both intra-assay and interassay precision. The procedure will remain consistent across all treatment groups. Once analysis is complete, blood samples will be discarded. Heart rate will be recorded as beat-to-beat intervals using Polar H10 sensors with chest straps and the Elite HRV application on Android phones at 1000 Hz.^54^,^56^ Beat-to-beat intervals will measure the time between successive R-spikes. To analyse HRV indices such as RMSSD (‘Heart Rate Variability’, 1996), we will record at least 5 min of heart rate activity while patients are sitting. Data will be collected and documented through an electronic CRF (eCRF/EDC-System) hosted by KKS Marburg, with secure web-based data entry (HTTPS (TSL/SSL) and supplementary outcome information, including any complications.

### Data analysis plan

The hypotheses of primary interest refer to the feasibility of the study design and acceptability of the iEXPECT intervention as an internet-based intervention, which will be assessed based on three criteria:

1. Feasibility of the study design is assumed if at least one participant per week per centre can be recruited (approximately 160 participants from three centres over 53 weeks), and >49% of all randomised patients provide complete data for the 6-month follow-up assessments, including biomarkers. The recruitment threshold of ≥1 participant per centre per week was based on the assumption that a Phase III trial with *X* centres could achieve its enrolment target within a recruitment period of *Y* under this rate. The ≥49% randomisation threshold was chosen to reflect an acceptable screening-to-randomisation efficiency while accounting for expected attrition. The proportion of patients with complete data for the 6-month follow-up assessments (including biomarkers) will be reported as a point estimate per group. Two-sided 95% CIs will be given.
2. Feasibility of the internet-based intervention is assumed if >75% of the randomised patients in each iEXPECT arm complete at least 1 (>0) online module before the surgery. The completion rate will be reported as a point estimate per intervention group, and two-sided 95% CIs will be provided. To assess whether time to surgery influences outcome criterion 2 (completion of at least one online module before surgery), a sensitivity analysis will be considered.
3. Acceptance will be assessed by self-report ratings at the end of each online module and with the CSQ-I after the intervention (T1) and the 6 months follow-up (T3). To evaluate a session’s/the intervention’s enjoyableness, usefulness and effect on daily social functioning using a 5-point scale (1=fully disagree, 2=disagree, 3=not sure, 4=agree, 5=fully agree; examples for items: ‘This session/intervention was useful and sensible’, ‘I would recommend this session/intervention to others’, ‘This session/intervention lived up to my expectations’). Acceptance of the internet-based intervention is assumed if the mean rating for overall acceptance/satisfaction with the intervention (calculated after completion of each of the four modules) exceeds 3.4. This threshold was chosen to reflect a tendency toward agreement rather than neutrality (>3=‘not sure’) and is supported by prior data^12^ suggesting an expected mean of 3.7 (SEM=0.14), making >3.4 a conservative and statistically robust criterion. Each intervention group will be analysed separately and mean rating of acceptance/satisfaction with the intervention will be presented for each online module. An overall mean rating across the four modules/predefined timepoints of assessment will be calculated.

Baseline characteristics will be presented by group. For categorical variables, the number of patients per category will be reported as well as the category proportion. The number of missing values will also be presented. For continuous variables, mean and SD will be reported. Primary and secondary outcome variables will be analysed in the same way at the predefined timepoints. Secondary outcomes will be analysed descriptively to identify relevant trends, inform intervention refinement and guide power calculations for a future Phase III trial. While the study is not powered to detect efficacy, exploratory correlation and regression analyses may be performed to examine relationships among secondary outcome variables and to generate hypotheses for subsequent confirmatory research.

### Sample size and study power

This feasibility study will explore the rate of completing at least one online module before surgery (outcome criterion 2). This will provide valuable information on what can be expected in a future Phase III trial. As one proportion, CIs are used to estimate a population proportion, sample size calculation is based on a CI approach. For a future Phase III trial, we want to assure that at least 75% of the patients in an iEXPECT group will complete at least one online module before surgery. In our study, we expect a completion rate of 84% in each intervention group, because it provides a meaningful buffer above the 75% feasibility threshold and ensures that the lower bound of the 95% CI will remain above this criterion, while keeping the required sample size feasible. Using a two-sided 95% CI with a lower CI limit of 75%, and an expected completion rate of 84%, we found that each intervention group should have at least 64 patients for the study. As the primary focus lies on assessing the feasibility of the online interventions, the calculated sample size is required for each of the two intervention groups. Since this is a feasibility trial and not designed to detect statistically significant differences between groups, we decided to allocate only half as many participants (N=32) to the SOC group. We consider this sample size sufficient to provide preliminary insights into secondary outcomes within the SOC group. Accordingly, patients will be randomised in a 2:2:1 ratio across the two intervention arms (iEXPECT enhanced: iEXPECT limited) and the SOC arm, resulting in a total sample size of N=160. The sample size calculation was performed using PASS V.14 software (NCSS; www.ncss.com).

### Data monitoring

To ensure confidentiality, all study data will be deidentified using a unique coding system (e.g., first patient in Marburg ‘176-001’), with personal identifiers replaced by anonymised codes. Only study investigators will have access to the coded dataset. Results will be published exclusively in anonymised form. As part of quality assurance, a random sample of entries will undergo source document verification; for example, surgical dates in REDCap will be cross-checked against patient medical records to confirm accuracy. The study management team maintains a subject identification list within REDCap, enabling targeted verification in collaboration with clinical sites. While the study is ongoing, access to the data is strictly limited to the investigators, and all output will comply with the Consolidated Standards of Reporting Trials 2025 Statement guidelines. Data analysis and manuscript preparation will be carried out jointly by the study statisticians and investigators, followed by submission to peer-reviewed journals and presentation at international conferences. Participants may request study results following their involvement.

### The Independent Data Monitoring Committee

The Independent Data Monitoring Committee (IDMC), comprising two experts in medical psychology and e-mental health interventions, is responsible for independently evaluating patient safety in the clinical trial. The IDMC receives regular updates in case of serious adverse events, including suicide attempts or suicides, and addresses safety concerns. It conducts regular assessments of data integrity and validity, monitors the overall conduct of the clinical trial and provides recommendations. Importantly, IDMC members operate independently from the coordinating investigators and participating centres. As this study is a feasibility trial with a smaller sample size and no dedicated funding for an IDMC, two members were deemed sufficient for the committee, compared with three in the larger PSY-HEART-II trial.^57^ While the inclusion of clinical trialists, statisticians and consumer representatives is planned for the subsequent full-scale study, the current IDMC ensures appropriate methodological and clinical oversight within the scope and resource constraints of this preliminary phase.

### Patient and public involvement

While there was no formal patient and public involvement (PPI) in the design, conduct or dissemination of the iHEART trial, patient perspectives were indirectly integrated through prior studies, clinical experience and close collaboration with cardiac surgeons. The iEXPECT intervention builds on earlier face-to-face formats, which incorporated patient feedback—such as increasing the number of booster sessions and undergoing pilot testing with patient volunteers. However, an important aspect of the current iHEART trial is to gather patient feedback on the acceptability of specific intervention modules, which will directly inform the refinement and optimisation of the intervention in a subsequent full-scale Phase III trial. For future studies, we intend to explicitly incorporate PPI, drawing on the quantitative and qualitative findings from the iHEART trial and potentially involving organisations such as the German Heart Foundation to support and guide the development process more systematically.

## Ethics and dissemination

### Ethics

The research involving human participants underwent review and received approval from the Ethics Committee of the Department of Medicine at Philipps University of Marburg (Approval Number: AZ 229/23 BO; Date: 8 November 2023) and the Ethics Committee of the Department of Medicine at the University of Giessen (Approval Number: AZ 186/23 Date: 5 February 2024) for the study centres in Giessen and Bad Nauheim. All patients will voluntarily provide written informed consent to participate in the study. The protocol conforms to the Declaration of Helsinki, ensuring that patients receive standard medical procedures. Any modifications to the protocol require amendments in consultation with both Ethics Committees and will be communicated to investigators and participants. Detailed written patient information and consent are provided in the online supplemental appendix. Patients can contact the study management at any time during and after the study period. The trial will end once the target sample size is reached.

Patients who decline participation at specific assessment time points will still be contacted at later time points, where appropriate, to explore reasons for withdrawal and gather relevant follow-up information. Patients in the iEXPECT condition may decline individual conversations but will continue participation without any changes. Any deviations from the protocol will be recorded, and adverse events will be documented and reported immediately to the principal investigators. The principal investigators will evaluate these adverse events and provide guidance on how to handle them.

### Dissemination

Study findings will be disseminated to participants, the public, policy-makers and the scientific community via peer-reviewed publications and conference presentations. Furthermore, we aim to compose a text that is comprehensible to laypersons and make it available to relevant consumer advocacy groups (e.g., the German Heart Foundation) for potential publication on their website, thereby reaching the public.

### Data availability statement

Data will be made available on reasonable request following the completion of the study and finalisation of data collection.

## Discussion

This study aims to assess the feasibility of the study design at various locations and the acceptability of iEXPECT, an online adaptation from the traditional face-to-face ‘EXPECT’ intervention. It will explore two variations of the online intervention with different levels of therapist support. This feasibility trial serves as a precursor to a larger future Phase III trial that will evaluate the efficacy of the iEXPECT intervention.

### Feasibility

We anticipate high feasibility for the study as patients are not required to attend additional appointments at the clinic prior to hospital admission. In the ongoing PSY-HEART-II trial, we recruited approximately 110 patients over 12 months using ten centres. However, recruitment was significantly hindered by two waves of the COVID-19 crisis, which led to the postponement of all elective surgeries. The iHEART trial only necessitates that patients have regular access to a computer with an internet connection and a valid email, thus avoiding additional travel costs and patient journeys. The only extra effort required from patients is the time needed to complete the questionnaires and the intervention. Booster emails (iEXPECT limited) or phone calls (iEXPECT enhanced) are scheduled at 6, 12 and 18 weeks after patients return home. We plan to recruit approximately 160 patients in 12 months (plus a 6-month follow-up) with three participating centres, given that the recruitment process will not be complicated by the COVID-19 crisis.

### Digital technology use by older adults

When considering the utilisation of online interventions for preoperative optimisation of treatment expectations in the older adults’ population, several factors merit attention. First, digital literacy among older individuals varies widely,^58^ potentially impacting their ability to navigate internet-based platforms effectively. Moreover, issues such as limited access to stable internet connections and disparities in device ownership (e.g., computers, headphones) may pose barriers to participation. The Technology Acceptance Model (TAM) provides a framework for understanding the acceptance of new technologies, suggesting that perceived usefulness and ease of use influence adoption rates.^59 60^ Recent work has indicated that there exists a prevailing prejudice against internet-based interventions among older adults, stemming from unfamiliarity with digital technologies and scepticism regarding their efficacy.^61^ Furthermore, compared to face-to-face interventions, online approaches may require significant investment in human contact to facilitate user engagement and address potential technical challenges,^62 63^ underscoring the importance of tailored support and guidance throughout the intervention process.

Therefore, conducting a feasibility study is crucial to assess the viability of such interventions in this demographic. Furthermore, testing different forms of guidance can provide insights into what works best (online guidance vs personal contact via telephone calls) for older adult individuals, addressing concerns related to digital literacy, internet connection issues, device ownership bias, prejudice against internet-based interventions, unfamiliarity and the TAM in older adults.

## Conclusion

The iHEART trial aims to demonstrate the potential of a scalable internet-based intervention, that if comparable in feasibility and acceptance to the face-to-face approaches, could significantly enhance healthcare access for cardiac surgery patients. If successful, it could also serve as a model for other medical conditions. For instance, meta-analytical evidence emphasises the relationship of preoperative expectations and surgery outcome in elective surgeries such as total knee and total hip arthroplasty.^64 65^ Positive outcomes from the feasibility study could signify a valuable step forward, paving the way for a larger multicentric effectiveness study if iHEART proves feasible and acceptable.

## Supplementary material

10.1136/bmjopen-2024-092482 online supplemental file 1

10.1136/bmjopen-2024-092482 online supplemental file 2
