# Supplementary material for: iHEART trial: study protocol for a German multicentre randomised controlled trial on the feasibility and acceptance of an internet-based preoperative intervention to optimise patient expectations and improve outcomes after heart surgery
Source: BMJ Open. 2025 Sep 17;15(9):e092482. doi: 10.1136/bmjopen-2024-092482 (PMC12458749; doi:10.1136/bmjopen-2024-092482)
Supplement: online supplemental file 1 [file bmjopen-15-9-s001.docx]

Supplementary Material

**Table S1.** **World Health Organization trial registration data set.**

| **Items** | **Information** |
| --- | --- |
| Primary registry and trial identifying number | www.drks.de, DRKS00033284 |
| Date of registration in primary registry | 22.12.2023 |
| Secondary identifying numbers | SA 4505/3-1 - DFG |
| Source(s) of monetary or material support | German Research Foundation (“Deutsche Forschungsgemeinschaft”, DFG) |
| Primary sponsor | Philipps University of Marburg |
| Secondary sponsor(s) | Department for Cardiovascular Surgery, Heart Center, Philipps University of Marburg, Marburg, Germany;  Department for Cardiovascular Surgery, Justus-Liebig-University, Giessen, Germany.  Department of Cardiac Surgery, Heart Center, Kerckhoff-Klinik GmbH Bad Nauheim  German Research Foundation (“Deutsche Forschungsgemeinschaft”, DFG) |
| Contact for public queries | Prof. Dr. Stefan Salzmann, Philipps University of Marburg, Division of Clinical Psychology and Psychotherapy, Gutenbergstraße 18, 35032 Marburg, Germany; telephone: +4964212823350; email: [stefan.salzmann@staff.uni-marburg.de](mailto:stefan.salzmann@staff.uni-marburg.de)  Division of Medical Psychology, HMU Health and Medical University, Am Anger 64-73, 99084 Erfurt, Germany; telephone: +49 151-22314813; email: stefan.salzmann@hmu-erfurt.de |
| Contact for scientific queries | Prof. Dr. Stefan Salzmann, Philipps University of Marburg, Division of Clinical Psychology and Psychotherapy, Gutenbergstraße 18, 35032 Marburg, Germany; telephone: +4964212823350; email: [stefan.salzmann@staff.uni-marburg.de](mailto:stefan.salzmann@staff.uni-marburg.de)  Division of Medical Psychology, HMU Health and Medical University, Am Anger 64-73, 99084 Erfurt, Germany; telephone: +49 151-22314813; email: stefan.salzmann@hmu-erfurt.de |
| Public title | Feasibility and acceptance of an internet-based preoperative intervention optimizing patients’ expectations to improve outcome in heart surgery patients (the iHEART trial) |
| Scientific title | The iHEART Trial: Study Protocol for a German Multicenter Randomized Controlled Trial on the Feasibility and Acceptance of an Internet-Based Preoperative Intervention to Optimize Patient Expectations and Improve Outcomes After Heart Surgery |
| Countries of recruitment | Germany |
| Health condition(s) or problem(s) studied | I25 - Chronic ischaemic heart disease |
| Intervention(s) | Arm 1, Intervention name: iEXPECT + enhanced guidance (iEXPECT enhanced)  Intervention description: guided preoperative psychological online intervention, which includes providing feedback via telephone calls after every completed module, aims to optimize expectations. |
|  | Arm 2, Intervention name: iEXPECT + limited guidance (iEXPECT limited)  Intervention description: guided preoperative psychological online intervention, which includes providing feedback via emails after every completed module, aims to optimize expectations. |
|  | Control group: Standard of care (SOC), no additional treatment. |
| Key inclusion and exclusion criteria | Ages eligible for study: ≥ 18 years.  Sexes eligible for study: both.  Accepts healthy volunteers: no. |
|  | Inclusion criteria: Medical necessity to perform a CABG (without valve), elective surgery, median sternotomy, with/without ECC, sufficient knowledge of the German language, Adequate cognitive fitness to give consent to participate in the study, Valid email address, Regular access to a computer with an internet connection, Consent at least 5 to 21 days before the surgery. |
|  | Exclusion criteria: Emergency surgery, minimally invasive surgical procedure, presence of another (non-cardiac) life-threatening condition, comorbid medical/psychiatric condition causing at least a similar impairment compared to coronary and valvular heart disease, participation in other interventional/experimental research programs. |
| Study type | Feasibility |
|  | Method of allocation: randomized. |
|  | Masking: medical staff (caregiver, treating physician) |
|  | Assignment: three-arm (2:2:1).  Randomization: permuted block randomization; Prestratifications: study center (Marburg, Giessen or Bad Nauheim); and the baseline value of disease-related impairment (Pain Disability Index (PDI), <23 or >=23). |
|  | Primary purpose: Feasibility and Acceptance of a Guided Preoperative Internet-based Expectation Manipulation Intervention (iEXPECT). |
| Date of first enrolment | 08.11.2024 |
| Target sample size | 160 |
| Recruitment status | Recruiting. |
| Primary outcome(s) | 1. Feasibility of the study design is assumed if at least one participant per week per center can be recruited (approximately 160 participants from three centers over 53 weeks), and >49% of all randomized patients provide complete data for the 6-month follow-up assessments, including biomarkers. 2. Feasibility of the internet-based intervention is assumed if >75% of the randomized patients in each iEXPECT arm complete at least 1 (>0) online module before the surgery. 3. Acceptability is assessed via self-reported ratings of enjoyableness, usefulness, and perceived impact on daily functioning after each module with acceptance defined as a mean score >3.4 on a 5-point Likert scale and using the CSQ-I, to assess interest and satisfaction in the iEXPECT intervention after completion and before the surgery (at T1) and six months follow up (T3). |
| Key secondary outcomes | Illness-related disability, expectations and prior experiences, subjective illness representations, health-related quality of life, preoperative anxiety, anxiety, depression, physical activity, optimism, pain, perceived stress, beliefs about online interventions, digital health literacy, side effects, social support, rehospitalization, length of stay, length of intensive care unit stay, planned and realized surgery, adverse events, satisfaction with intervention and medical treatment, inflammatory markers, heart rate variability (HRV), EURO-SCORE II, NYHA, LVEF, blood pressure, CCS score, smoking, BMI. |
| Ethics review | Status: Approved.  1. Ethics Committee of the Department of Medicine of the Philipps University of Marburg (AZ 229/23 BO; Date: 2023-11-08)  2. Ethics Committee of the Department of Medicine of the University of Giessen (AZ 186/23 Date: 2024-02-05). |
| Completion date | Pending. |
| Summary results | Pending. |
| IPD sharing statement | Data can be shared on demand. |

Note. World Health Organization trial registration data set version 3.6. Timepoint (T), after intervention/pre-surgery (T1), 6 months post-surgery (T3); CSQ-I, Client Satisfaction Questionnaire [1], PDI, pain disability index [2].

**Table S2. Overview of assessment**

| Measurement | T0: Baseline | T1: preoperatively on the day of hospital admission | T2: seven days post-surgery | T3: 6-month follow-up |
| --- | --- | --- | --- | --- |
| Questionnaires | | | | |
| Illness-related disability (PDI, adapted version) | ✓ | ✓ | ✓ | ✓ |
| Quality of Life (SF-12); | ✓ | ✓ | ✓ | ✓ |
| Preoperative anxiety and need for information (APAIS) | ✓ | ✓ | ✓ | ✓ |
| Anxiety and depression (PHQ9 und GAD-7) | ✓ | ✓ | ✓ | ✓ |
| Pain ratings (VAS) | ✓ | ✓ | ✓ | ✓ |
| Trait optimism (LOT-R) | ✓ | ✓ | ✓ | ✓ |
| Perceived stress (PSS) | ✓ | ✓ | ✓ | ✓ |
| The International Physical Activity Questionnaire (IPAQ) | ✓ | ✓ | ✓ | ✓ |
| Attitudes towards Psychological Online Interventions (APOI) | ✓ |  |  |  |
| Subjective illness beliefs (B-IPQ) | ✓ | ✓ | ✓ | ✓ |
| Pre-treatment experiences, Treatment Expectations, and Treatment Effects (G-EEE) | ✓ | ✓ | ✓ | ✓ |
| Treatment expectations (TEX- Q) | ✓ | ✓ | ✓ | ✓ |
| Treatment satisfaction (i.e., rating of fulfilment/violation of pre-treatment expectations at the end of the trial) |  |  |  | ✓ |
| Mechanisms of interventions (e.g., outcome expectations, personal control, coping) | ✓ | ✓ | ✓ | ✓ |
| Social support (ESSI) | ✓ | ✓ |  |  |
| Digital Health Literacy (GR-eHEALS) | ✓ |  |  |  |
| Side-effects (NEQ) |  | ✓ |  |  |
| Demographics (e.g., age, gender) | ✓ |  |  |  |
| Acceptance, interest in/satisfaction with the intervention (CSQ-I) |  | ✓ |  | ✓ |
| Experience with previous surgeries | ✓ |  |  |  |
| Biological Parameters | | | | |
| Immune parameters (CRP, IL-6) | ✓ | ✓ | ✓ | ✓ |
| HRV (i.e., triangular index) | ✓ | ✓ | ✓ | ✓ |
| Case report form (CRF) | | | | |
| LVEF | ✓ | ✓ | ✓ | ✓ |
| Blood pressure | ✓ | ✓ | ✓ | ✓ |
| BMI | ✓ |  |  |  |
| Smoking | ✓ |  |  |  |
| EuroSCORE II | ✓ |  |  |  |
| NYHA status | ✓ |  |  |  |
| CCS score | ✓ |  |  |  |
| Planned surgical procedure | ✓ |  |  |  |
| Realized surgical procedure |  |  | ✓ |  |
| Previous surgeries | ✓ |  |  |  |
| Medication | ✓ |  |  |  |
| Clinical data (e.g., duration of surgery, hospital stay, Intensive Care Unit) |  |  | ✓ | ✓ |
| Rehospitalization, and serious medical events during follow-up; |  |  |  | ✓ |
| Complications |  |  | ✓ | ✓ |
| Interview (screening) |  |  |  |  |
| SCID-I | ✓ |  |  |  |

Note. APAIS, Amsterdam preoperative anxiety and information scale [3, 4]; APOI, attitudes towards psychological online interventions [5]; B-IPQ, brief-illness-perception questionnaire [6]; BMI, body mass index; CCS, Canadian Cardiovascular Society [7]; CRP, C-reactive protein; CSQ-I, Client Satisfaction Questionnaire [1]; ESSI-D, enriched social support inventory-deutsch [8]; EuroSCORE, European system for cardiac operative risk evaluation [9]; GAD-7, generalized anxiety disorder scale [10]; G-EEE, generic rating scale for previous treatment experiences, treatment expectations, and treatment effects [11]; GR-eHEALS, revised German ehealth literacy scale [12]; HRV, heart-rate-variability; IL-6, Interleukin-6, IL-8, Interleukin-8; IPAQ, international physical activity questionnaire [13]; LVEF, left ventricular ejection fraction; LOT-R, revised life orientation test [14]; New York heart association (NYHA) score; NEQ, negative effects questionnaire [15]; PDI, pain disability index [2]; PHQ-9, patient health questionnaire [9]; PSS, Perceived Stress Scale [16, 17]; SF-12, short-form health survey [18] SCID, structured clinical interview for DSM-IV [19]; TEX-Q, treatment expectation questionnaire [20]; VAS, visual analogue scale.Bottom of Form

Bottom of Form

References

1. Boß L, Lehr D, Reis D, Vis C, Riper H, Berking M, et al. Reliability and Validity of Assessing User Satisfaction With Web-Based Health Interventions. J Med Internet Res. 2016;18:e234.
2. Tait RC, Chibnall JT, Krause S. The Pain Disability Index: psychometric properties. Pain. 1990;40:171–82.
3. Moerman N, van Dam FS, Muller MJ, Oosting H. The Amsterdam Preoperative Anxiety and Information Scale (APAIS). Anesth Analg. 1996;82:445–51.
4. Berth H, Petrowski K, Balck F. The Amsterdam Preoperative Anxiety and Information Scale (APAIS) - the first trial of a German version. Psychosoc Med. 2007;4:Doc01.
5. Schröder J, Sautier L, Kriston L, Berger T, Meyer B, Späth C, et al. Development of a questionnaire measuring Attitudes towards Psychological Online Interventions-the APOI. J Affect Disord. 2015;187:136–41.
6. Broadbent E, Petrie KJ, Main J, Weinman J. The brief illness perception questionnaire. Journal of psychosomatic research. 2006;60:631–7.
7. Kaul P, Naylor CD, Armstrong PW, Mark DB, Theroux P, Dagenais GR. Assessment of activity status and survival according to the Canadian Cardiovascular Society angina classification. Can J Cardiol. 2009;25:e225–3
8. Hoskings JD. Enhancing recovery in coronary heart disease patients (ENRICHD): Study design and methods. American Heart Journal. 2000;139 1 I:1–9.
9. Johansson M, Nozohoor S, Zindovic I, Nilsson J, Kimblad PO, Sjögren J. Prediction of 30-day mortality after transcatheter aortic valve implantation: a comparison of logistic EuroSCORE, STS score, and EuroSCORE II. The Journal of heart valve disease. 2014;23:567–74.
10. Löwe B, Kroenke K, Herzog W, Gräfe K. Measuring depression outcome with a brief self-report instrument: sensitivity to change of the Patient Health Questionnaire (PHQ-9). Journal of Affective Disorders. 2004;81:61–6.
11. Rief W, Nestoriuc Y, Mueller EM, Hermann C, Schmidt K, Bingel U. Generic rating scale for previous treatment experiences , treatment expectations , and treatment effects ( GEEE ) Generic rating scale for previous treatment experiences , treatment expectations , and treatment effects ( GEEE ) University of Duisburg-Esse. 2021; April. https://doi.org/10.23668/psycharchives.4717.
12. Marsall M, Engelmann G, Skoda E-M, Teufel M, Bäuerle A. Measuring Electronic Health Literacy: Development, Validation, and Test of Measurement Invariance of a Revised German Version of the eHealth Literacy Scale. J Med Internet Res. 2022;24:e28252.
13. Craig CL, Marshall AL, Sjöström M, Bauman AE, Booth ML, Ainsworth BE, et al. International physical activity questionnaire: 12-country reliability and validity. Medicine and science in sports and exercise. 2003;35:1381–95.
14. Glaesmer H, Hoyer J, Klotsche J, Yorck Herzberg P. Die deutsche Version des Life-Orientation-Tests (LOT-R) zum dispositionellen Optimismus und Pessimismus. Zeitschrift für Gesundheitspsychologie. 2008;32:26–31.
15. Rozental A, Kottorp A, Forsström D, Månsson K, Boettcher J, Andersson G, et al. The Negative Effects Questionnaire: psychometric properties of an instrument for assessing negative effects in psychological treatments. Behav Cogn Psychother. 2019;47:559–72.
16. Cohen S, Kamarck T, Mermelstein R. A global measure of perceived stress. J Health Soc Behav. 1983;24:385–96.
17. Klein EM, Brähler E, Dreier M, Reinecke L, Müller KW, Schmutzer G, et al. The German version of the Perceived Stress Scale – psychometric characteristics in a representative German community sample. BMC Psychiatry. 2016;:1–10.
18. Ware J, Kosinski M, Keller SD. A 12-Item Short-Form Health Survey: construction of scales and preliminary tests of reliability and validity. Medical care. 1996;34:220–33.
19. First MB, Spitzer RL, Gibbon M, Williams JBW. Structured Clinical Interview for DSM-IV Axis I Disorders, Clinician Version (SCID-CV). Washington, DC: American Psychiatric Press; 1996.
20. Alberts J, Löwe B, Glahn MA, Petrie K, Laferton J, Nestoriuc Y, et al. Development of the generic, multidimensional Treatment Expectation Questionnaire (TEX-Q) through systematic literature review, expert surveys and qualitative interviews. BMJ Open. 2020;10.
