# Supplementary material for: iHEART trial: study protocol for a German multicentre randomised controlled trial on the feasibility and acceptance of an internet-based preoperative intervention to optimise patient expectations and improve outcomes after heart surgery
Source: BMJ Open. 2025 Sep 17;15(9):e092482. doi: 10.1136/bmjopen-2024-092482 (PMC12458749; doi:10.1136/bmjopen-2024-092482)
Supplement: online supplemental file 2 [file bmjopen-15-9-s002.docx]

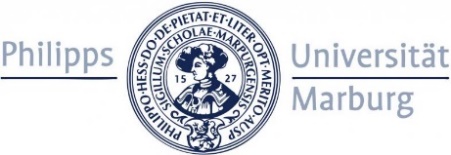

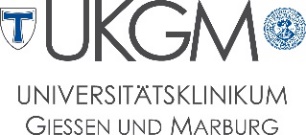


**Studienleitung:**

Prof. Dr. A. Rastan

Prof. Dr. S. Salzmann

**Ansprechpartnerin:** Dr. S. Balci

**Tel:** 06421 28 23623

**Mail:** iHEART@uni-marburg.de

**Patienteninformation**

**Machbarkeit einer internetbasierten Operationsvorbereitung**

**vor herzchirurgischen Eingriffen (iHeart)**

Sehr geehrte Patientin, sehr geehrter Patient,

wir möchten Sie dazu einladen an der nachfolgend beschriebenen klinischen Studie **„Individuelle internetbasierte Operationsvorbereitung vor herzchirurgischen Eingriffen (iHEART-Studie)“** teilzunehmen. Bitte nehmen Sie sich einen Moment Zeit, um sich über die Studie informieren zu lassen, die an unserer Klinik durchgeführt wird. Bitte lesen Sie diese Informationen sorgfältig durch, damit Sie entscheiden können, ob Sie teilnehmen möchten oder nicht. Sollten noch Fragen offen sein, können Sie uns über folgendes Kontaktformular [hier wird ein Link für ein Kontaktformular eingefügt, auf welches Patient:innen klicken können] eine Telefonnummer oder eine Emailadresse hinterlassen, um Sie kontaktieren zu können. Unser Team steht Ihnen für Fragen zur Verfügung und wird diese beantworten. Danach werden Sie ausreichend Bedenkzeit erhalten, um über eine Teilnahme zu entscheiden. Wenn Sie an der Studie teilnehmen möchten, bitten wir Sie Ihre Einwilligung zur Teilnahme mit dem Setzen eines Kreuzes (am Ende des Dokuments) zu bestätigen. Damit bestätigen Sie, dass Sie die vorgelegten Informationen vollständig verstanden und der Teilnahme an dieser Studie zugestimmt haben. Mit Ihrer Teilnahme an dieser Studie helfen Sie herauszufinden, ob diese „Individuelle internetbasierte Operationsvorbereitung vor herzchirurgischen Eingriffen“ als Internetintervention angewendet kann und die Behandlung von Patient:innen bei Herzoperationen zu verbessern.

**Hintergrund zu der Studie**

Diese klinische Studie wird an mehreren Kliniken (3) in Deutschland durchgeführt; es sollen insgesamt 160 Personen teilnehmen. Die Studie wird durch die Philipps-Universität Marburg (Klinik für Herz- und thorakale Gefäßchirurgie des Herzzentrums, Arbeitsgruppe Klinische Psychologie und Psychotherapie und dem Koordinierungszentrum für Klinische Studien der Philipps-Universität Marburg) organisiert. Die Finanzierung erfolgt durch die Deutsche Forschungsgemeinschaft (DFG).

**Was ist das Ziel der Studie?**

Die persönliche Sicht der eigenen Herzerkrankung kann für den Genesungsprozess eine wichtige Rolle spielen. Eine individuelle Vorbereitung kann Betroffene im Verlauf einer Herzoperation in Ihrem Genesungsprozess unterstützen. Im Rahmen der Studie bieten wir Ihnen eine ergänzende internetbasierte individuelle Operationsvorbereitung an. Außerdem möchten wir Sie über Ihr Krankheitserleben und Ihre Erwartungen bezüglich der geplanten Herzoperation befragen, um Sie individuell und bestmöglich auf die bevorstehende Herzoperation vorzubereiten.

In einer Vorstudie wurde diese „Individuelle Operationsvorbereitung“ im persönlichen Kontakt angewendet und von Patienten/ Patientinnen wie Ihnen bereits sehr gut angenommen und als hilfreich für den Genesungsprozess eingeschätzt. Dennoch konnte trotz großem Interesse und hoher Nachfrage ein großer Teil an Patienten/ Patientinnen nicht an der Intervention teilnehmen. Grund dafür waren verschiedene Barrieren wie beispielsweise eine zu lange Anreise für zusätzliche Termine im Krankenhaus oder zu wenig Personal um die Intervention durchzuführen. Das Ziel dieser Studie ist es daher zu untersuchen, ob diese „Individuelle Operationsvorbereitung“ auch in internetbasierter Form angewendet werden kann. Mit Hilfe eines internetbasierten Angebots können gleichzeitig viele Personen unterstützt werden und man kann sich bereits zu Hause zu einem selbst gewählten Zeitpunkt auf die Operation und den Genesungsprozess vorbereiten.

**Wie läuft die Studie ab?**

Vor Ihrer Herzoperation werden Sie in jedem Fall im Rahmen der standardisierten Patientenaufklärung ausführlich von dem Stationsarzt/ der Stationsärztin bzw. dem Operateur/ der Operateurin über das genaue operative Vorgehen und die möglichen Risiken einer Operation aufgeklärt. Dies ist unabhängig von Ihrer Studienteilnahme.

Im Rahmen der Studie erhalten Sie die Möglichkeit, entweder die normale medizinische Standardversorgung oder zusätzlich zur medizinischen Standardversorgung eine von zwei internetbasierten geführten individuellen Operationsvorbereitungen (mit personalisierter Rückmeldung) durch einen Studientherapeuten/ eine Studientherapeutin wahrzunehmen. Die beiden Angebote unterscheiden sich zwar in der Form der Rückmeldung (per Telefon oder Email), haben aber beide zum Ziel, Sie bei der optimalen Bewältigung Ihrer Herzoperation zu unterstützen. Es wird per Zufall entschieden, ob und falls ja, welche der beiden zusätzlichen Angebote Sie erhalten. Die Wahrscheinlichkeit eine der beiden Operationsangebote zu erhalten beträgt ca. 80% (ca. 40% Wahrscheinlichkeit für jedes einzelne Angebot), während eine Wahrscheinlichkeit von 20% besteht, dass Sie ausschließlich die medizinische Standardversorgung erhalten. Die zusätzlichen Angebote bestehen jeweils aus vier Online-Modulen, die Sie von Zuhause am PC durchführen können und vier persönlichen Rückmeldungen zu den in den Modulen behandelten Inhalten (per Telefon oder Email) vor der Operation sowie drei Booster-Module zur Auffrischung (pro Online Module jeweils eine Rückmeldung) 6, 12 und 18 Wochen nach der Operation.

Das erste Online-Modul (Dauer ca. 20-40 Min.) findet (frühestens) etwa 10 Tage vor der Operation statt. Die Module können in eigener Zeit durchgearbeitet werden und sollten bis spätestens ein Tag vor der Herz-OP beendet (in der Regel der Aufnahmetag in die Klinik) sein. Die personalisierte Rückmeldung erhalten Sie jeweils nach Beendigung eines Moduls (1-4) entweder per Email oder es wird mit Ihnen ein Telefontermin vereinbart, zu dem sich der Studientherapeut/ die Studientherapeutin bei Ihnen melden wird (Dauer etwa 10-15 min). Etwa 6, 12 und 18 Wochen nach der Operation kann ein weiteres Online Modul durchgearbeitet werden zur Auffrischung der zuvor erarbeiteten Inhalte kombiniert mit einer persönlichen Rückmeldung (per Telefon ca. 10-15 min oder Email).

Es besteht die Möglichkeit, dass Sie gleichzeitig an anderen klinischen Studien teilnehmen, falls jene nicht mit dieser Studie konkurrieren. Bitte teilen Sie uns dies mit, falls Sie an anderen Studien teilnehmen. Dann wird im Einzelfall durch das Studienteam entschieden, ob die Teilnahme an dieser Studie möglich ist.

**Wer kann an der Studie teilnehmen?**

Sie können an der „iHEART“-Studie teilnehmen, wenn:

- Sie sich Ihrer ersten geplanten aortokoronaren Bypass-Operation (CABG, ohne Klappeneingriff) mit oder ohne Einsatz der Herzlungenmaschine unterziehen
- Bei Ihnen eine mediane Sternotomie durchgeführt wird
- Sie ausreichende Kenntnisse der deutschen Sprache besitzen
- Ausreichende kognitive Fitness um Einverständnis zur Studienteilnahme zu geben
- Sie eine gültige Email Adresse haben
- Regelmäßigen Zugang zu einem Computer mit Internetanschluss haben
- Sich mindestens 5 bis 21 Tage vor der OP befinden
- Sie über 18 Jahre alt sind

**Wer kann nicht teilnehmen?**

Sie können nicht an der „iHEART“-Studie teilnehmen, wenn:

- Sie sich einer Notfalloperation unterziehen
- Ihre Operation ein minimal-invasiver chirurgischer Eingriff ist
- Bei Ihnen ein Eingriff an einer Herzklappe vorgesehen ist
- Vorhandensein eines anderen (nicht kardialen) lebensbedrohlichen Zustands
- Sie an einer anderen körperlichen Erkrankung oder psychischen Störung leiden, die Sie in Ihrem Leben stärker beeinträchtigt als Ihre Herzerkrankung
- Sie an anderen interventionellen oder experimentellen Forschungsprogrammen teilnehmen, die die Ergebnisse unserer Studie beeinflussen könnten.

**Was ist der persönliche Nutzen einer Teilnahme?**

Wenn Sie sich für die Studienteilnahme entscheiden, nehmen Sie mit einer Wahrscheinlichkeit von 80% an einer der beiden individuellen Operationsvorbereitungen teil. Das Angebot soll Sie dabei unterstützen, mit den Belastungen der Operation besser umzugehen und sich danach schneller zu erholen. Darüber hinaus leisten Sie einen wichtigen Beitrag zur wissenschaftlichen Forschung und tragen dazu bei, dass die medizinische Versorgung mit Hilfe von Studien wie dieser weiter verbessert wird. Auch für den Fall, dass Sie im Rahmen der Studie keine zusätzliche internetbasierte psychologische Vorbereitung erhalten, ist Ihre Teilnahme sehr wichtig. Nur wenn wir nach Ende der Datenerhebung Personen vergleichen, die eine zusätzliche Vorbereitung erhalten haben mit jenen, die keine erhalten haben, können wir feststellen welchen Effekt die zusätzliche Intervention hat.

**Was bedeutet die Teilnahme an der Studie für Sie persönlich?**

Nachdem Sie Ihr Einverständnis gegeben haben kommt folgender Ablauf auf Sie zu:

- Wenn Sie eingewilligt haben an der Studie teilzunehmen (und Ihre Daten wie Namen, Geburtsdatum, Email und Telefonnummer angegeben haben) ordnen wir Ihnen eine sogenannte Patienten-Nummer oder auch Patienten-ID (abgekürzt Pat.-ID) zu. Diese Pat.-ID dient dazu, dass alle weiteren Daten von Ihnen im Verlauf der Studie nur in Verbindung mit dieser Pat.-ID gespeichert werden (und nicht mit Ihrem Klarnamen) damit nur wir die Daten den jeweiligen Patient:innen zuordnen können. Dieses Vorgehen nennt man Pseudonymisierung (das wird weiter unten nochmal ausführlicher erklärt).
- Nachdem Sie schriftlich in die Teilnahme an der Studie eingewilligt haben, werden wir Sie telefonisch kontaktieren. In diesem persönlichen Gespräch mit einem/r Arzt/Ärztin oder einem/r Psycholog:in, werden wir nochmals auf wichtige Inhalte aus der Aufklärung eingehen, um sicherstellen zu können, dass Sie alles richtig verstanden haben. Zum Beispiel werden wir nochmals die Ein- und Ausschlusskriterien abklären, auf die mit der Blutentnahme verbundenen Risiken eingehen sowie noch offene Fragen zum Ablauf klären. Sofern Sie dann auch weiterhin mit einer Studienteilnahme einverstanden sind, werden wir ein kurzes Interview mit Ihnen durchzuführen, in dem wir Sie nach psychischen Beschwerden fragen (Dauer etwa 45 Min).
- Im nächsten Schritt erhalten Sie per Email einen Link zu einem Fragebogen, den Sie online unter Angabe Ihrer Pat.-ID (diese teilen wir Ihnen mit) ausfüllen sollen. Die Fragebogendatenerhebungen finden online über die Unipark-Plattform (www.unipark.de) statt (Dauer ca. 45 min). In dieser Email finden Sie auch eine ausführliche Anleitung wie das funktioniert. Im Zweifel unterstützen wir Sie auch gerne telefonisch.
- Sobald Sie den Fragebogen abgeschlossen haben, werden Sie nochmals kontaktiert. Es wird mit Ihnen ein Termin vereinbart, zu dem unsere Study Nurse/ Studienschwester Sie bei Ihnen zu Hause besucht, um Ihnen Blut abzunehmen und Ihre Herzaktivität zu messen. Es werden Blutproben (3 mal 7,5) entnommen, die verwendet werden um zu bestimmen, wie hoch bestimmte Entzündungswerte (C-reaktives Protein, Interleukin-6, Interleukin-8) in Ihrem Blut sind. Ihre Herzaktivität wird für ca. 5 Minuten abgeleitet, um die sogenannte Herzratenvariabilität bestimmen zu können.
- Nach diesen ersten Untersuchungen werden Sie durch unser Studienteam per Zufall auf eine der drei Studienarme verteilt. Sofern Sie in einer der beiden Studienarme mit zusätzlicher internetbasierter OP-Vorbereitung landen, werden wir Ihnen einen weiteren Link per Email zukommen lassen, über den Sie Zugang zu der Online-Plattform (minddistrict) für die OP-Vorbereitung erhalten. Über diese Plattform können Sie dann die einzelnen Module bearbeiten und erhalten von uns – je nach Bedingung – jeweils Rückmeldung per Telefon oder per Email. Nach der Operation durchlaufen Patient:innen in den Studienarmen mit zusätzlicher Vorbereitung noch drei weitere sogenannten Booster-Module.
- Neben den Fragebogendaten und den biologischen Daten (Blutproben, Herzaktivität) werden Informationen aus Ihrer Patientenakte (Medikation, Vorerkrankungen, Herzfunktion, Blutdruck, Größe, Gewicht, Informationen zur durchgeführten Operation wie z.B. die Länge der Operation, Komplikationen, Anzahl der verwendeten Grafts, Aufenthaltsdauer auf der Intensivstation, Länge des Krankenhausaufenthalts) entnommen und elektronisch mit Ihrer Pat.-ID gespeichert.

Zu drei weiteren Zeitpunkten werden diese Erhebungen wiederholt:

- Nach dem Aufklärungsprozess und den Gesprächen am Aufnahmetag in der Klinik bzw. vor der OP (Fragebögen und biologische Daten werden im Krankenhaus erhoben)
- Etwa 7 Tage nach der Operation (Fragebögen und biologische Daten werden im Krankenhaus erhoben)
- 6 Monate nach der Operation (wie zum ersten Messzeitpunkt werden Ihre Fragebögen online erhoben und die biologischen Daten durch einen Besuch bei Ihnen zu Hause erfasst)

Die nachfolgenden Befragungen sind kürzer als die erste und umfassen jeweils das Ausfüllen eines Fragebogens (ca. 30 Min.), die Blutprobenentnahme und die Bestimmung der Herzratenvariabilität. Über den gesamten Studienablauf können Sie Kontakt zu unserem Studienteam aufnehmen (z.B. über iHEART@uni-marburg.de). Wir unterstützen Sie gerne.

Falls innerhalb der Gespräche mit den Projekmitarbeitern/ der Projekmitarbeiterinnen (Arzt/ Ärztin oder Studientherapeut/ Studientherapeutin) Zufallsbefunde, wie z.B. psychische Störungen diagnostiziert werden, können Sie darüber entscheiden, wie mit diesen umgegangen werden soll, d.h. ob Sie über diese in Kenntnis gesetzt werden möchten oder nicht.

Hierfür kreuzen Sie bitte im Falle einer Einwilligung zur Studienteilnahme das entsprechende Kästchen an (siehe weiter unten).

**Mögliche Nachteile und Risiken**

Medizinisch sind außer der Blutentnahme keine Eingriffe mit dieser Studie verbunden. Bei der Blutentnahme wird venöses Blut entnommen (3 x 7,5 ml). Durch die Blutentnahme kann die Haut an der Einstichstelle schmerzen oder anschwellen und an der Einstichstelle kann sich ein Bluterguss bilden. In extrem seltenen Fällen kann es zu Nervenschädigungen kommen. Die Blutproben werden zur Analyse versandt und anschließend vernichtet. Zur Bestimmung Ihrer Herzaktivität werden Elektroden auf Brust und Bauch aufgeklebt und das Signal mit Hilfe eines mobilen Messgeräts erfasst. Daher sind außer dem Zeitaufwand keine Nachteile durch die Studienteilnahme zu erwarten.

**Besteht während der Studienteilnahme eine Versicherung?**

Während der Teilnahme an dem Forschungsprojekt genießen Sie einen Versicherungsschutz, um die zusätzlichen Blutentnahmen (zu Studienbeginn und 6 Monate nach OP) abzusichern, da diese außerhalb der regulären Standardversorgung studienbedingt durchgeführt wird. Der Umfang des Versicherungsschutzes ergibt sich aus den Versicherungsunterlagen.

Wenn Sie vermuten, dass durch die Teilnahme an der Studie Ihre Gesundheit geschädigt oder bestehende Leiden verstärkt wurden, müssen Sie dies unverzüglich dem Versicherer direkt anzeigen, gegebenenfalls mit Unterstützung durch Ihren/r Studientherapeut/ Studientherapeutin oder Arzt/ Ärztin, um Ihren Versicherungsschutz nicht zu gefährden. Sofern Ihr/e Studientherapeut/ Studientherapeutin oder Ihr/e Arzt/ Ärztin Sie dabei unterstützt, erhalten Sie eine Kopie der Meldung.

Name und Anschrift der Versicherung:

QBE Europe SA/NV

Direktion für Deutschland

Breite Str. 31

40213 Düsseldorf

Versicherungsnummer: LS01 0000053

Kundennummer: 2330 99 1104 / 1965

Sofern Sie Ihre Anzeige direkt an den Versicherer richten, informieren Sie bitte zusätzlich Ihre/n Studientherapeut/ Studientherapeutin oder Arzt/ Ärztin.

Bei der Aufklärung der Ursache oder des Umfangs eines Schadens müssen Sie mitwirken und alles unternehmen, um den Schaden abzuwenden und zu mindern.

**Datenschutz und Freiwilligkeit**

Die Teilnahme an der Studie ist freiwillig. Der Rücktritt von Ihrem Einverständnis zur Teilnahme ist jederzeit ohne Angabe von Gründen möglich und mit keinerlei Nachteilen für Sie verbunden.

Während der klinischen Studie werden medizinische Befunde und persönliche Informationen von Ihnen erhoben und in der Klinik in Ihrer persönlichen Studienakte niedergeschrieben bzw. elektronisch gespeichert. Die für die klinische Prüfung wichtigen Daten werden zusätzlich in pseudonymisierter Form gespeichert, ausgewertet und gegebenenfalls an das Studienzentrum in Marburg und das Koordinierungszentrum für Klinische Studien der Universität Marburg weitergegeben. Im Rahmen von Maßnahmen zur Qualitätssicherung kann es vorkommen, dass vom Auftraggeber der Studie autorisierte und zur Verschwiegenheit verpflichtete Personen Einblick in Ihre Patientenunterlagen nehmen. Es wird zugesichert, dass alle Beteiligten die erhaltenen Informationen vertraulich behandeln.

Bei der Pseudonymisierung (Verschlüsselung) werden der Name und andere Identifikationsmerkmale (z.B. Teile des Geburtsdatums) durch z.B. eine mehrstellige Buchstaben- oder Zahlenkombination, auch Code genannt, ersetzt, um die Identifizierung des Studienteilnehmenden wesentlich zu erschweren. Die Zuordnung zwischen Ihrem Namen, Kontaktdaten und Pat.-ID wird ausschließlich in einem verschlüsselten Excel-Dokument auf dem Server der AG Klinische Psychologie und Psychotherapie, Fachbereich Psychologie der Uni Marburg gespeichert. Zugang zu diesem Ordner auf dem Server bzw. zu dem „Schlüssel“, der eine persönliche Zuordnung Ihrer Daten zu Ihrem Namen ermöglicht, haben neben Ihrem/r behandelnde/n Arzt/ Ärztin und Ihrem/r Studientherapeut/in nur von diesem/r ausdrücklich autorisierte Personen aus Ihrem Studienzentrum sowie die jeweiligen Projektmitarbeiter: innen. In den weiteren Schritten werden für alle weiteren Datenerhebungen nur die jeweiligen Pat-IDs verwendet, sodass alle Daten in pseudonymisierter Form vorliegen und kein Rückschluss auf die Identität der Personen möglich ist. Sobald der Forschungszweck es zulässt, spätestens jedoch zum 31.12. 2028, wird der Schlüssel gelöscht und die erhobenen Daten damit anonymisiert. Ihre Daten werden ausschließlich für Auswertungen im Rahmen der Studie verwendet. Die Auswertung und Nutzung der Daten erfolgen in pseudonymisierter Form. Die Veröffentlichung der Studienergebnisse erfolgt ausschließlich in anonymisierter Form. Alle Projektmitarbeiter/ Projektmitarbeiterinnen unterliegen der Schweigepflicht. Damit die Studientherapeut/ Studientherapeutinnen der Arbeitsgruppe Klinische Psychologie und Psychotherapie mit den Ärzten/ Ärztinnen des Herzzentrums und dem Koordinierungszentrum für Klinische Studien der Universität Marburg Rücksprache halten können, ist es notwendig, dass Sie uns von der Schweigepflicht entbinden. Ihr Einverständnis kann jederzeit widerrufen werden.

Jederzeit ist es für Sie möglich, Auskunft über Ihre gespeicherten personenbezogenen Daten zu verlangen. Außerdem haben Sie das Recht, fehlerhafte Daten zu berichtigen, Daten löschen zu lassen oder zu jeder Zeit Ihre Einwilligung zur Verarbeitung Ihrer personenbezogenen Daten zu widerrufen. Bitte setzen Sie sich hierfür mit dem verantwortlichen Studienleiter in Kontakt bzw. kontaktieren Sie die angegebene Ansprechpartnerin.

Im Falle des Widerrufs der Einwilligungserklärung zur Teilnahme an der klinischen Studie dürfen die bis zu diesem Zeitpunkt gespeicherten Daten weiterhin verwendet werden, sofern Sie diesem Vorgehen nicht widersprechen. Alle Stellen, die Ihre personenbezogenen Daten, insbesondere Gesundheitsdaten, gespeichert haben, müssen unverzüglich prüfen, inwieweit die gespeicherten Daten noch erforderlich sind. Nicht mehr benötigte Daten sind unverzüglich zu löschen. Eine Löschung bereits anonymisierter Daten ist nicht möglich.

Sollten Sie noch weitere Fragen zu unserem Projekt haben, zögern Sie nicht, uns zu kontaktieren.

**Einwilligung**

**Bitte wählen Sie nun eine der drei folgenden Optionen aus:**

- **Ich habe noch Fragen und möchte diese gerne mit dem Studienteam besprechen indem das Team Kontakt mit mir aufnimmt.**

Bitte füllen Sie folgende Felder aus, damit wir mit Ihnen Kontakt aufnehmen können:

Optional: Name

Obligatorisch: Email-Adresse:___________ und/oder Telefonnummer:__________

[Sofern Patient:innen diese Auswahl treffen, werden Sie von uns kontaktiert und bestehende

Fragen geklärt. Wenn die Patient:innen dann an der Studie teilnehmen möchten, werden Sie

gebeten wieder den ursprünglichen Link zur Pat-Info aufzurufen und Option 3 auszuwählen]

- **Ich möchte NICHT an der Studie teilnehmen.**

[Sofern Patient:innen diese Auswahl treffen, werden Sie auf eine weitere Website geleitet, auf der Ihnen für Ihre bisheriges Interesses gedankt wird]

- **Ich möchte an der Studie teilnehmen.** Ich habe die Patienteninformation gelesen und verstanden. Ich hatte ausreichend Gelegenheit meine Fragen zu klären und über die Durchführung der klinischen Studie zu sprechen. Alle meine Fragen wurden zufriedenstellend beantwortet. Ich hatte ausreichend Zeit, mich zu entscheiden. Mir ist bekannt, dass ich jederzeit und ohne Angabe von Gründen meine Einwilligung zur Teilnahme an der klinischen Studie zurückziehen kann (mündlich oder schriftlich), ohne dass mir daraus Nachteile jedweder Art entstehen.

Im Anschluss an Ihre Zustimmung zur Teilnahme werden wir Sie nochmal persönlich kontaktieren. In diesem persönlichen Gespräch mit einem/r Arzt/Ärztin oder einem/r Psycholog:in, werden wir nochmals auf wichtige Inhalte aus der Aufklärung eingehen, um sicherstellen zu können, dass Sie alles richtig verstanden haben. In diesem Gespräch haben Sie ebenfalls die Möglichkeit noch offene Fragen zu klären.

Bitte füllen Sie noch folgende Felder aus:

Name:__________________

Vorname:_______________

Geburtsdatum:___________

Email-Adresse:___________

Telefonnummer:__________

Wir bitten Sie um Ihre Zustimmung, dass wir Daten aus Ihrer Patientenakte für Studienzwecke entnehmen dürfen (z.B. Medikamente, Informationen zu Ihrer Operation, eine umfassendere Auflistung der relevanten Informationen finden Sie weiter oben) und elektronisch mit Ihrer Pat.-ID gespeichert:

Einwilligung Zufallsbefunde:

- Ja, ich stimme zu, dass im Rahmen der Studie Daten aus meiner Patientenakte entnommen werden dürfen.
- Nein, ich stimme nicht zu, dass im Rahmen der Studie Daten aus meiner Patientakte entnommen werden dürfen.

Bitte geben Sie uns noch eine Rückmeldung wie wir bei Ihnen mit Zufallsbefunden umgehen sollen, sofern diese auftreten:

Einwilligung Zufallsbefunde:

- Ja, ich möchte über Zufallsbefunde, die im Rahmen der Studie diagnostiziert werden, in Kenntnis gesetzt werden.
- Nein, ich möchte nicht über Zufallsbefunde, die im Rahmen der Studie diagnostiziert werden, in Kenntnis gesetzt werden und mache vom meinem Recht auf Nichtwissen Gebrauch.

Die folgenden beiden Dokumente können jeweils über einen Link auf der Aufklärungsseite aufgerufen werden:

|  | **Datenschutz**  **(Dokument kann über einen Link im:** |
| --- | --- |
|  |  |
|  | Mir ist bekannt, dass bei dieser klinischen Studie personenbezogene Daten, insbesondere medizinische Befunde über mich erhoben, gespeichert und ausgewertet werden sollen. Die Verwendung der Daten erfolgt nach gesetzlichen Bestimmungen und setzt vor der Teilnahme an der klinischen Studie folgende freiwillig abgegebene Einwilligungserklärung voraus, das heißt, ohne die nachfolgende Einwilligung kann ich nicht an der klinischen Studie teilnehmen.  1. Ich erkläre mich damit einverstanden, dass im Rahmen dieser klinischen Studie personenbezogene Daten, insbesondere Angaben über meine Gesundheit erhoben und in Papierform sowie auf elektronischen Datenträgern in der Prüfstelle aufgezeichnet werden. Soweit erforderlich, dürfen die erhobenen Daten pseudonymisiert (verschlüsselt) weitergegeben werden:  a) an das Koordinierungszentrum für Klinische Studien Marburg der Philipps Universität Marburg und an die AG Klinische Psychologie und Psychotherapie der Philipps Universität Marburg zur wissenschaftlichen Auswertung. Sowie an der Studie beteiligte Forscher, welche einen Teil der Daten getrennt zum Zwecke der wissenschaftlichen Forschung auswerten.  b) im Falle unerwünschter Ereignisse: an das Koordinierungszentrum für Klinische Studien Marburg der Philipps Universität Marburg, an die jeweils zuständige Ethik- Kommission, an die AG Klinische Psychologie und Psychotherapie der Philipps Universität Marburg.  2. Außerdem erkläre ich mich damit einverstanden, dass autorisierte und zur Verschwiegenheit verpflichtete Beauftragte der Studienleitung in meine bei den behandelnden Ärzten/ Ärztinnen vorhandenen personenbezogenen Daten, insbesondere meine Gesundheitsdaten, Einsicht nehmen, soweit dies für die Überprüfung der ordnungsgemäßen Durchführung der klinischen Studie notwendig ist. Für diese Maßnahme entbinde ich die behandelnden Ärzte/ Ärztinnen von der ärztlichen Schweigepflicht.  3. Ich bin darüber aufgeklärt worden, dass ich jederzeit die Teilnahme an der klinischen Studie beenden kann. Ich weiß, dass im Falle eines Widerrufs zur Teilnahme an der klinischen Studie die bis zu diesem Zeitpunkt gespeicherten Daten nur weiterhin verwendet werden dürfen, soweit dies erforderlich ist, um sicherzustellen, dass meine schutzwürdigen Interessen nicht beeinträchtigt werden.  4. Ich erkläre mich damit einverstanden, dass meine Daten nach Beendigung oder Abbruch der klinischen Studie mindestens zehn Jahre aufbewahrt werden. Danach werden meine personenbezogenen Daten gelöscht, soweit nicht gesetzliche, satzungsmäßige oder vertragliche Aufbewahrungsfristen entgegenstehen.  5. Ich bin über folgende gesetzliche Regelung informiert: Falls ich meine Einwilligung an der  klinischen Studie teilzunehmen, widerrufe, müssen alle Stellen, die meine |

personenbezogenen Daten, insbesondere Gesundheitsdaten, gespeichert haben, unverzüglich prüfen, inwieweit die gespeicherten Daten noch erforderlich sind. Nicht mehr benötigte Daten sind unverzüglich zu löschen.

7. Ich bin über meine in der Datenschutz-Grundverordnung (DS-GVO) festgelegten Rechte

informiert worden (anhand Anlage 1 „Information für Studienteilnehmer gemäß
 Europäischer Datenschutz-Grundverordnung (gültig ab 25.05.2018) für
 medizinische Forschungsvorhaben.

**Information für Studienteilnehmer gemäß**

**für medizinische Forschungsvorhaben**

**1**

Machbarkeit einer internetbasierten Operationsvorbereitung

vor herzchirurgischen Eingriffen (iHeart)

**Hiermit möchten wir Sie über die in der Datenschutz-Grundverordnung (= DS-GVO)** **festgelegten Rechte informieren** (Artikel 12 ff. DS-GVO):

**Rechtsgrundlage**

Die Rechtsgrundlage zur Verarbeitung der Sie betreffenden personenbezogenen Daten bilden bei klinischen Studien (einschließlich klinischer Prüfungen) Ihre freiwillige schriftliche Einwilligung gemäß DS-GVO sowie der Deklaration von Helsinki (Erklärung des Weltärztebundes zu den ethischen Grundsätzen für die medizinische Forschung am Menschen) und der Leitlinie für Gute Klinische Praxis. Bei Arzneimittel-Studien ist zusätzlich das Arzneimittelgesetz, bei Medizinprodukte-Studien das Medizinproduktegesetz anzuwenden.

**Bezüglich Ihrer Daten haben Sie folgende Rechte** (Artikel 13 ff. DS-GVO):

**Recht auf Auskunft**

Sie haben das Recht auf Auskunft über die Sie betreffenden personenbezogenen Daten, die im Rahmen der klinischen Studie erhoben, verarbeitet oder ggf. an Dritte übermittelt werden (Aushändigen einer *kostenfreien* Kopie) (Artikel 15 DS-GVO).

**Recht auf Berichtigung**

Sie haben das Recht Sie betreffende unrichtige personenbezogene Daten berichtigen zu lassen (Artikel 16 und 19 DS-GVO).

**Recht auf Löschung**

Sie haben das Recht auf Löschung Sie betreffender personenbezogener Daten, z.B. wenn diese Daten für den Zweck, für den Sie erhoben wurden, nicht mehr notwendig sind oder Sie Ihre Einwilligung widerrufen, auf die sich die Verarbeitung Ihrer Daten stützt. Die Rechtmäßigkeit der aufgrund der Einwilligung bis zum Widerruf erfolgten Verarbeitung wird hiervon nicht berührt." (Artikel 7, 17 und 19 DS-GVO).

**Recht auf Einschränkung der Verarbeitung**

Unter bestimmten Voraussetzungen haben Sie das Recht, die Einschränkung der Verarbeitung zu verlangen, d.h. die Daten dürfen nur gespeichert, aber nicht verarbeitet werden. Dies müssen Sie beantragen. Wenden Sie sich hierzu bitte an Ihren Prüfer oder an den Datenschutzbeauftragten des Prüfzentrums (Artikel 18 und 19 DS-GVO).

**Im Falle der Berichtigung, Löschung, Einschränkung der Verarbeitung** werden zudem all jene benachrichtigt, die Ihre Daten erhalten haben (Artikel 17 Absatz 2 und Artikel 19 DS-GVO).

**Recht auf Datenübertragbarkeit**

Sie haben das Recht, die Sie betreffenden personenbezogenen Daten, die Sie dem Verantwortlichen für die klinische Studie bereitgestellt haben, zu erhalten. Damit können Sie beantragen, dass diese Daten entweder Ihnen oder, soweit technisch möglich, einer anderen von Ihnen benannten Stelle übermittelt werden (Artikel 20 DS-GVO).

**Widerspruchsrecht**

Sie haben das Recht, bei Vorliegen von Gründen, die sich aus Ihrer besonderen Situation ergeben, jederzeit gegen konkrete Entscheidungen oder Maßnahmen zur Verarbeitung der Sie betreffenden personenbezogenen Daten Widerspruch einzulegen (Artikel 21 DS-GVO). Eine solche Verarbeitung findet anschließend grundsätzlich nicht mehr statt.

**Einwilligung zur Verarbeitung personenbezogener Daten und Recht auf Widerruf dieser** **Einwilligung**

Die Verarbeitung Ihrer personenbezogenen Daten ist mit Ihrer Einwilligung rechtmäßig (Artikel 6 DS-GVO). Sie haben das Recht, Ihre Einwilligung zur Verarbeitung personenbezogener Daten jederzeit zu widerrufen. Die Rechtmäßigkeit der aufgrund der Einwilligung bis zum Widerruf erfolgten Verarbeitung wird hiervon nicht berührt (Artikel 7 Absatz 3 DS-GVO).

**Benachrichtigung bei Verletzung des Schutzes personenbezogener Daten** **(„Datenschutzpannen“)**

Hat eine Verletzung des Schutzes personenbezogener Daten voraussichtlich ein hohes Risiko für Ihre persönlichen Rechte und Freiheiten zur Folge, so werden Sie unverzüglich benachrichtigt (Artikel 34 DS-GVO).

**Übermittlungen personenbezogener Daten an Drittländer oder an internationale** **Organisationen**

**[Die Übermittlung personenbezogener Daten an Drittländer ist nicht geplant.]**

*Bezug auf Artikel 44-50 DS-GVO*

**Möchten Sie eines dieser Rechte in Anspruch nehmen, wenden Sie sich bitte an** Ihren Prüfer oder an den Datenschutzbeauftragten Ihres Prüfzentrums. Außerdem haben Sie das **Recht, Beschwerde bei der/den Aufsichtsbehörde/n einzulegen,** wenn Sie der Ansicht sind, dass die Verarbeitung der Sie betreffenden personenbezogenen Daten gegen die DS- GVO verstößt (**siehe Kontaktdaten**).

**Datenschutz: Kontaktdaten des Sponsors/der Studienleitung der klinischen Studie in** **Marburg**

| **Datenschutzbeauftragte/r** | | **Datenschutz-Aufsichtsbehörde** | |
| --- | --- | --- | --- |
| Name: | Prof. Dr. Stefan Salzmann | Name: Der Hessische Datenschutz-  beauftragte |  |
| Adresse: | Gutenbergstraße 18  35037 Marburg | Adresse: | Gustav-Stresemann-Ring 1  65189 Wiesbaden |
| Telefon: | 06421-28 23350 | Telefon: | 0611-140 80 |
| Fax: | 06421-28-28904 | Fax: | 0611-140 8611 |
| E-Mail: | stefan.salzmann@uni-marburg.de | E-Mail | poststelle@datenschutz.hessen.de |
| Internet: | https://www.uni-marburg.de | Internet: | https://datenschutz.hessen.de/servic  e/beschwerde |

| **Für die Datenverarbeitung Verantwortliche/r** | |
| --- | --- |
| ggf. Name | Prof. Dr. Stefan Salzmann |
| Adresse: | Gutenbergstraße 18  35037 Marburg |
| Telefon: | 06421-28 23350 |
| Fax: | 06421-28-28904 |
| E-Mail | stefan.salzmann@uni-marburg.de |
| Internet: | https://www.uni-marburg.de |
