# Supplementary material for: iHEART trial: study protocol for a German multicentre randomised controlled trial on the feasibility and acceptance of an internet-based preoperative intervention to optimise patient expectations and improve outcomes after heart surgery
Source: BMJ Open. 2025 Sep 17;15(9):e092482. doi: 10.1136/bmjopen-2024-092482 (PMC12458749; doi:10.1136/bmjopen-2024-092482)
Supplement: Supplementary file 3 [file bmjopen-2024-092482.reviewer_comments.pdf]

PEER REVIEW HISTORY

BMJ Open publishes all reviews undertaken for accepted manuscripts. Reviewers are asked to complete a checklist review form and are provided with free text boxes to elaborate on their assessment. These free text comments are reproduced below.

ARTICLE DETAILS

Title (Provisional)

The iHEART Trial: Study Protocol for a German Multicenter Randomized Controlled Trial on the Feasibility and Acceptance of an Internet-Based Preoperative Intervention to Optimize Patient Expectations and Improve Outcomes After Heart Surgery

Authors

Compère, Viviane Noelani; Balci, Sümeyye; Heinz, Christine; Schade-Brittinger, Carmen; Rastan, Ardawan J.; Salzmann-Djufri, Miriam; Niemann, Bernd; Boening, Andreas; Choi, Yeong-Hoon; Zarski, Anna-Carlotta; Laferton, Johannes; Euteneuer, Frank; Rief, Winfried; Salzmann, Stefan

---

VERSION 1 - REVIEW

**Reviewer**	**1**
**Name**	**Codispodi, Kelly**
**Affiliation**	**University of Manitoba, Physical Therapy**
**Date**	**30-Apr-2025**
**COI**	**None**

There is a conflicting statement in line 8 on pg 5 (pg # per footer), about the pilot informing a future multi-center efficacy trial, however line 32 on pg 7 identifies this trial to be a multicenter RCT. Please correct or elaborate on the expansion to include more centers in future.

For your consideration, post op collection of biomarkers at 7 days may be challenging if patients are discharged earlier - I would suggest collection at 7 days post op or discharge from hospital, if earlier. This will likely increase feasibility as you won't require patients to return for bloodwork and HRV assessment if discharged prior to day 7.

**Reviewer**	**2**
**Name**	**Gibbons, Kristen**
**Affiliation**	**The University of Queensland, Child Health Research Centre**

**Date**	**16-May-2025**
**COI**	**None**

Thank you for the opportunity to review the iHEART study protocol. This pilot trial examines an interesting topic. While there are a number of clarifications required to enable the reader to fully understand the study methodology, the study has merit. Please find my detailed comments below.

ABSTRACT

1. This study appears to pilot trial, however this is not stated in the Abstract. Please include reference to the study being a pilot.

2. Consider rewording the second sentence of the Methods section; it is confusing whether group 1 receives SOC plus additional psychological intervention, or if the additional psychological intervention refers only to groups 2 and 3.

3. Please include additional detail on what the EXPECT intervention is. It is noted that there are online modules and the timepoints explained, but the actual content of the intervention is not provided. Is the EXPECT online modules the additional psychological intervention, or is the psychological intervention the 'personal guidance'?

4. Consider providing more detail on the metrics examined for the primary outcomes (feasibility and acceptability) – this is currently provided for secondary outcomes for psychological outcomes, but the primary outcome metrics are not provided.

5. Consistent terminology is suggested – endpoints or outcomes

6. There is no dissemination approach outlined; please include.

STRENGTHS AND LIMITATIONS

1. The first point, describing the development of the intervention, is not part of this study – that is prior work. Please remove.

2. Is the acronym iHeart or iHEART – be consistent throughout (e.g. title uses iHEART but this section uses iHeart).

3. The wording of the third bullet point is hard to follow; consider revising this sentence for clarity, or split into two points, as it is addressing two concepts.

4. Please explicitly note that the last bullet point is a limitation.

INTRODUCTION

1. The study aim is listed as "…whether the traditional face-to-face "EXPECT" intervention for expectation optimization can be successfully adapted to an online format (iHEART trial)." However, I think the aim is more nuanced; this aim can be interpreted as the development

process for the online intervention, not the assessment of the intervention. I would ask the study team to reconsider the wording of this aim.

2. It's a bit confusing to have the EXPECT acronym used for both the original (non-digital) intervention and the digital intervention used in this trial. Could the researchers consider amending the intervention acronym to make it clearer for the reader when the digital version is being referred to?

3. The secondary outcomes listed in the last paragraph don't align with those in the Abstract; I would suggest that closer alignment would be preferable.

MATERIALS AND ANALYSIS

1. In Table 1, I would suggest that when describing the two interventions, it should be indicated that they are digital interventions. For study type, 'feasibility' is listed, but in the aims, the trial is referred to as a pilot. Please be consistent throughout. Please provide more granular detail on the primary outcomes – how is feasibility and acceptability being measured? Rather than 'Remaining', consider terminology such as 'Pending'.

2. Under Study Design, in the first sentence describing the trial, please include 'pilot'. In the same sentence, please include any relevant references to descriptions of the EXPECT intervention.

3. 'The trial guidance occurs…' could be interpreted as oversight of the trial (e.g. Steering Group), however this appears to relate to guidance provided to the participant as part of the intervention, not trial oversight. Please reword this statement to provide greater clarity it is associated with the intervention, not trial oversight. In the same paragraph, please consider amending 'Data will be collected with…' to a phrase that is more indicative that the recruitment is occurring at the listed centres as well.

4. Please describe why there was no patient and public involvement in trial design, conduct, or intended dissemination pathways. Given the type of intervention, PPI could have significantly contributed to higher chances of feasibility and acceptability.

5. Consider moving the 'Sample size and study power' paragraph to after the outcomes have been described; it is hard to interpret this paragraph without first learning about the study outcomes.

6. The sample size has been calculated on an outcome measure that is only relevant for two of the three study groups; this is highly unusual and does not align with demonstrating a difference between the three study groups. I would suggest that this choice of calculation is defended much more strongly, as I do not believe it is a valid approach to calculating sample size. If the current approach persists, please defend the choice of the 0.09 margin of error and the 75% lower limit. Additionally, why is 84% the expected completion rate? Please further defend how it was decided to half the number of participants in the control group; was this undertaken by PASS, or did the investigators extrapolate the 64/gp to the final sample size?

7. Is December 2025 when the last patient is expected to be recruited, or when the last follow-up occurs?

8. There appears to be a word missing in the last bullet point in the Inclusion Criteria – 5 'hours' or 5 'days' or 5 'months'?

9. When do the patients get approach for recruitment – how long before their surgery does this occur, and how long do they have to consider whether they will consent? Does the anonymous screening data get stored in the study database for both eligible and ineligible patients? Do ineligible patients receive any information about expectation setting? Who assesses the presence of relevant mental disorders as part of the screening process? If new mental disorders are identified, are there referral pathways to ensure appropriate care is provided for the patient?

10. What does 'PDI' refer to in the first sentence of 'Randomization'? Please spell out the abbreviation in full and reference it if appropriate. Who created the randomisation schedule and what is their relationship to the study? Are there back-up envelopes in case of technology failure? Are the participants instructed to conceal their randomisation from their treating medical team?

11. The pre-surgical assessments take place with quite a wide range (5-21 days before surgery); how will this wide window be handled during analysis?

12. Given the primary outcomes relate to feasibility and acceptability, do participants need to consent to blood sampling, or is that optional?

13. How long will the assessments take at each timepoint for the participant to complete? Will all assessment questionnaires be undertaken online, or a mixture of paper and online? What retention strategies are in place to encourage participants to complete all timepoints and all assessments at each timepoint?

14. Clarity of outcome measures for a clinical trial is extremely important. Presently, it is hard to identify all the outcome measures as they are dispersed between two sections and intermixed with data analysis approaches. Please amend the two outcome sections to purely describe the outcomes, identifying which are primary and which are secondary and including a definition for each.

15. Following, please include a paragraph on data analysis that includes details on the descriptive statistics that will be presented and what outcomes they will be presented for, any regression modelling that will be used and if so, what covariates will be included and what effect estimates and associated measure of variability will be presented, any subgroup or sensitivity analyses, howe missing data will be handled, how the temporal nature of the data will be incorporated in analyses, and how the type I error will be incorporated.

16. Please include a section on data monitoring – how will source data verification be undertaken. How will participant identifiability be guaranteed?

17. An IDMC of only two people seems small; please defend the size of this committee, and the lack of inclusion of clinical trialists/statisticians/consumers.

18. What reporting guideline will be used when reporting the study results?

ETHICS AND DISSEMINATION

1. Will participants be interviewed? It's unclear when and how this is occurring. If they are, please ensure this is clear in the Methods, including who is interviewing them, how the interview is recorded, how the transcript is generated, where the audio/video is stored, etc.

2. Please separate Ethics and Dissemination sections and place Ethics higher up in the Methods.

3. The Dissemination approach is very limited; please consider novel and consumer-informed methods of dissemination.

FIGURE

1. It cannot be known how many patients will be screened and how many will end up in each group; I would suggest removing any numbers from the figure.

VERSION 1 - AUTHOR RESPONSE

Reviewer #1

Dr. Kelly Codispodi, University of Manitoba

1. Comments to the Author: There is a conflicting statement in line 8 on pg 5 (pg # per footer), about the pilot informing a future multi-center efficacy trial, however line 32 on pg 7 identifies this trial to be a multicenter RCT. Please correct or elaborate on the expansion to include more centers in future.

Response:

- Thank you for your valuable feedback. We acknowledge that our wording may not have been sufficiently clear. Given that the primary objective of this study is to assess the feasibility of the design for a multi-center randomized controlled trial, we included multiple centers in the feasibility study to account for variability in hospital procedures and settings. This multi-center approach is intended to inform the planning and implementation of a subsequent larger trial aimed at assessing the efficacy of the iEXPECT intervention. We added to the 'Introduction' on p. 4 line 131-132: "Specifically, the primary objectives are to evaluate (1) the feasibility of implementing the study design across multiple sites, similar to the PSY-HEART-II trial (…)" and on p. 5 line 141-143: "This feasibility trial also serves as a precursor to a larger future study (phase III trial) aimed at evaluating the efficacy of the iEXPECT intervention. If successful, the iHEART trial could counteract the previous barriers and could thus improve access to better healthcare for patients undergoing heart surgeries."

2. For your consideration, post op collection of biomarkers at 7 days may be challenging if patients are discharged earlier - I would suggest collection at 7 days post op or discharge from hospital, if earlier. This will likely increase feasibility as you won't require patients to return for bloodwork and HRV assessment if discharged prior to day 7.

Response:

- Thank you for this valuable point. We will incorporate this recommendation into the study protocol to enhance feasibility and participant convenience. We have added on p. 10 line 351-353: "To enhance feasibility, postoperative collection of biomarkers will be scheduled for postoperative day 7 or at the time of hospital discharge, whichever occurs first, to avoid requiring patients to return for blood and HRV assessments after discharge."

Reviewer: 2

Dr. Kristen Gibbons, The University of Queensland

Comments to the Author: Thank you for the opportunity to review the iHEART study protocol. This pilot trial examines an interesting topic. While there are a number of clarifications required to enable the reader to fully understand the study methodology, the study has merit. Please find my detailed comments below.

Response:

- Thank you for this positive feedback.

ABSTRACT

1. This study appears to pilot trial, however this is not stated in the Abstract. Please include reference to the study being a pilot.

Response:

- Thanks for bringing our attention to this inconsistency. This study is a feasibility study that is meant to inform a future larger efficacy trial. We now consistently use "feasibility trial" to avoid misunderstandings.

2. Consider rewording the second sentence of the Methods section; it is confusing whether group 1 receives SOC plus additional psychological intervention, or if the additional psychological intervention refers only to groups 2 and 3.

Response:

- We have now rephrased the section to enhance clarity and ensure better understanding (p. 3, lines 63-66): "In this three-arm, multicenter randomized controlled trial (RCT), 160 patients undergoing elective coronary artery bypass graft (CABG) surgery are randomized to: (a) standard of care (SOC); (b) SOC plus iEXPECT with phone-based guidance (iEXPECT enhanced); or (c) SOC plus iEXPECT with email-based guidance (iEXPECT limited)."

3. Please include additional detail on what the EXPECT intervention is. It is noted that there are online modules and the timepoints explained, but the actual content of the intervention is not provided. Is the EXPECT online modules the additional psychological intervention, or is the psychological intervention the 'personal guidance'?

Response:

- We have added additional information what the intervention consists of on p.3 lines 66-69: "The intervention includes four 20-minute online modules addressing surgical benefits, side effects, and coping strategies. Modules are accompanied by personalized guidance provided

through feedback on each module via email or telephone (three before surgery, three booster sessions at 6, 12, and 18 weeks post-surgery)."

4. Consider providing more detail on the metrics examined for the primary outcomes (feasibility and acceptability) – this is currently provided for secondary outcomes for psychological outcomes, but the primary outcome metrics are not provided.

Response:

- We appreciate the reviewer's observation regarding the absence of clearly stated primary outcome metrics. We have now added the following on p.3 line 70-74 : "Primary feasibility outcomes include recruitment (≥1 participant/week/center), retention (≥49% completing 6-month follow-up including biomarkers), and engagement (≥75% completing ≥1 pre-surgery module). Acceptability is measured by self-reported enjoyment, usefulness, and impact, with acceptance defined as mean scores >3.4 (5-point Likert scale) and CSQ-I ratings."

5. Consistent terminology is suggested – endpoints or outcomes

Response:

- Thank you for bringing our attention to this inconsistency. We have decided to use the term "outcomes" throughout the manuscript for clarity and consistency and have adapted the text accordingly.

6. There is no dissemination approach outlined; please include.

Response:

- We have added the following text in the abstract (p. 3, lines 83-84): "Results will be shared via publications, conferences, and public outreach with potential support from the German Heart Foundation."

STRENGTHS AND LIMITATIONS

7. The first point, describing the development of the intervention, is not part of this study – that is prior work. Please remove.

Response:

- Done.

8. Is the acronym iHeart or iHEART – be consistent throughout (e.g. title uses iHEART but this section uses iHeart).

Response:

- Thanks for bringing our attention to this inconsistency. We have decided to use the term "iHEART" throughout the manuscript for clarity and consistency.

9. The wording of the third bullet point is hard to follow; consider revising this sentence for clarity, or split into two points, as it is addressing two concepts.

Response:

- We very much appreciate the feedback. We revised the 'Strengths and limitations of this study' section completely. The new bullet points are:

- Involving three diverse clinical centers enhances generalizability and feasibility assessment across different hospital settings.
- Use of stratified, permuted block randomization via REDCap ensures allocation concealment and minimizes selection bias.
- Treating physicians remain blinded to group assignment, reducing the risk of performance bias.
- Although previous patient feedback informed intervention development, there was no structured patient involvement in designing the current trial.
- Digital intervention format excludes patients without internet access or sufficient digital literacy, potentially limiting inclusivity.

10. Please explicitly note that the last bullet point is a limitation.

Response:

- Thank you for the feedback; we have revised the entire section accordingly—please refer to our response to point 9 above.

INTRODUCTION

11. The study aim is listed as "…whether the traditional face-to-face "EXPECT" intervention for expectation optimization can be successfully adapted to an online format (iHEART trial)." However, I think the aim is more nuanced; this aim can be interpreted as the development process for the online intervention, not the assessment of the intervention. I would ask the study team to reconsider the wording of this aim.

Response:

- We have revised the entire section accordingly on p. 4-5, lines 128-134: "In a previous step, the traditional face-to-face "EXPECT" intervention for expectation optimization was adapted into an online format (iEXPECT). The present study—the iHEART trial—aims to test the feasibility of the study design and the acceptability of the online, preoperative, guided psychological iEXPECT intervention. Specifically, the primary objectives are to evaluate (1) the feasibility of implementing the study design across multiple sites, similar to the PSY-HEART-II trial, (2) whether patients are able and willing to engage with the intervention as intended, and (3) the acceptability of the iEXPECT intervention from the patients' perspective."

12. It's a bit confusing to have the EXPECT acronym used for both the original (non-digital) intervention and the digital intervention used in this trial. Could the researchers consider amending the intervention acronym to make it clearer for the reader when the digital version is being referred to?

Response:

- We appreciate this valuable suggestion. In response, we have decided to designate the internet-adapted version of the EXPECT intervention as "iEXPECT."

13. The secondary outcomes listed in the last paragraph don't align with those in the Abstract; I would suggest that closer alignment would be preferable.

Response:

- Given the extensive range of secondary outcomes assessed and to avoid confusion and improve consistency, we now refer to them more broadly as *psychological measures* in the

abstract for clarity. This can be found in the abstract on p. 3, lines 75-76: "Secondary outcomes include psychological measures, inflammatory markers, and heart rate variability (HRV)." In the Introduction we amended on p. 5, lines 137-139: "Secondary outcomes include psychological and physiological measures that will be analyzed descriptively per group. Biomarkers will be collected to assess the feasibility of obtaining inflammatory markers and heart rate variability (HRV)."

MATERIALS AND ANALYSIS

14. In Table 1, I would suggest that when describing the two interventions, it should be indicated that they are digital interventions. For study type, 'feasibility' is listed, but in the aims, the trial is referred to as a pilot. Please be consistent throughout. Please provide more granular detail on the primary outcomes – how is feasibility and acceptability being measured? Rather than 'Remaining', consider terminology such as 'Pending'.

Response:

- Thank you for this suggestion. We have clarified that both interventions are digital formats of the iEXPECT intervention. This has been updated in the supplementary materials Table S1: "Intervention description: guided preoperative psychological online intervention, which includes providing feedback via telephone calls after every completed module, aims to optimize expectations."
- We have revised the manuscript to ensure consistent use of the term "feasibility trial" throughout. As explained in our response to comment 1, we clarified the rationale and terminology accordingly.
- Furthermore, we have now provided more detailed information on how feasibility and acceptability are being measured. These additions can be found supplementary materials Table S1: "Feasibility includes successful recruitment (≥1 participant per week per center) and retention (≥49% of all randomized patients completing 6-month follow-up assessments including biomarkers), as well as intervention engagement (≥75% of participants in each iEXPECT group completing at least one module pre-surgery). Acceptability is assessed via self-reported ratings of enjoyableness, usefulness, and perceived impact on daily functioning after each module with acceptance defined as a mean score >3.4 on a 5-point Likert scale and using the CSQ-I, to assess interest and satisfaction in the iEXPECT intervention at T1 and T3."
- We have revised the terminology accordingly and replaced "remaining" with "pending" to improve clarity. The change can be seen in the supplementary materials Table S1.

15. Under Study Design, in the first sentence describing the trial, please include 'pilot'. In the same sentence, please include any relevant references to descriptions of the EXPECT intervention.

Response

- Thank you for your suggestion. As noted in our response to comment 1, we refer to the study as a feasibility trial rather than a pilot trial to reflect its specific objectives and methodological focus. Regarding the EXPECT intervention, we have incorporated the relevant references by moving the descriptive paragraph from the "Intervention" section into the Study Design section. The revised version can now be found on p. 5, lines 153-160: "The content, derived from the face-to-face "EXPECT" intervention [11, 15, 20], aims to improve individual expectations, enhancing recovery for cardiac surgery patients through placebo effects [12, 14, 20]. Adapted to an online format, the intervention covers three main components to promote personalized, positive, realistic expectations and correct

dysfunctional beliefs: (1) benefit expectations of surgery, (2) personal control strategies (e.g., health behavior, lifestyle), and (3) coping with negative post-operative effects (see Table 1). These aspects of the intervention are expected to influence psychological factors and yield improvements such as reducing anxiety levels or depressive symptoms."

16. 'The trial guidance occurs…' could be interpreted as oversight of the trial (e.g. Steering Group), however this appears to relate to guidance provided to the participant as part of the intervention, not trial oversight. Please reword this statement to provide greater clarity it is associated with the intervention, not trial oversight. In the same paragraph, please consider amending 'Data will be collected with…' to a phrase that is more indicative that the recruitment is occurring at the listed centres as well.

Response:

- Thank you for this helpful clarification. We have reworded the sentence to avoid any misunderstanding and to clearly indicate that the guidance refers to participant support within the intervention. Furthermore, clarified that recruitment is also taking place at those centers**.** The revised section now reads: "As part of the intervention, guidance is provided through personalized feedback via phone calls (iEXPECT enhanced) or via email (iEXPECT limited). Participants will be recruited and data collected at the three participating centers: the university hospital of Marburg, the university hospital of Gießen, and the medical center Kerckhoff-clinic, Bad Nauheim. This change can be found on p. 5, lines 160-165.

17. Please describe why there was no patient and public involvement in trial design, conduct, or intended dissemination pathways. Given the type of intervention, PPI could have significantly contributed to higher chances of feasibility and acceptability.

Response:

- We have added an explanation for the absence of formal patient and public involvement (PPI) in the trial design, conduct, and dissemination. Although there was no structured PPI process, patient perspectives were indirectly incorporated through prior studies, clinical experience, and collaboration with cardiac surgeons. Future trials will explicitly include formal PPI components. We amended the text on p. 12 lines 449-458: "While there was no formal Patient and Public Involvement (PPI) in the design, conduct, or dissemination of the iHEART trial, patient perspectives were indirectly integrated through prior studies, clinical experience, and close collaboration with cardiac surgeons. The iEXPECT intervention builds on earlier face-to-face formats, which incorporated patient feedback—such as increasing the number of booster sessions and underwent pilot testing with patient volunteers. However, an important aspect of the current iHEART trial is to gather patient feedback on the acceptability of specific intervention modules, which will directly inform the refinement and optimization of the intervention in a subsequent full-scale phase III trial. For future studies, we intend to explicitly incorporate PPI, drawing on the quantitative and qualitative findings from the iHEART trial and potentially involving organizations such as the German Heart Foundation to support and guide the development process more systematically."

18. Consider moving the 'Sample size and study power' paragraph to after the outcomes have been described; it is hard to interpret this paragraph without first learning about the study outcomes.

Response:

- Done. The paragraph has now been moved to p. 11-12, lines 404-420.

19. The sample size has been calculated on an outcome measure that is only relevant for two of the three study groups; this is highly unusual and does not align with demonstrating a difference between the three study groups. I would suggest that this choice of calculation is defended much more strongly, as I do not believe it is a valid approach to calculating sample size. If the current approach persists, please defend the choice of the 0.09 margin of error and the 75% lower limit. Additionally, why is 84% the expected completion rate? Please further defend how it was decided to half the number of participants in the control group; was this undertaken by PASS, or did the investigators extrapolate the 64/gp to the final sample size?

Response:

- Thank you for this thoughtful and important comment. We would like to clarify and further justify our sample size calculation approach. As noted in the revised manuscript (p. 11-12, lines 404-420), this feasibility trial is not designed or powered to detect statistically significant differences between the three study arms. Rather, its primary aim is to assess the feasibility and acceptance of the two digital intervention formats, particularly regarding the rate of module completion prior to surgery. Accordingly, the sample size calculation was based on one of the predefined feasibility criteria—namely, the proportion of patients in the intervention groups who complete at least one online module before surgery (outcome criterion 2). To estimate this population parameter with sufficient precision, we applied a confidence interval-based approach, which is widely recommended for feasibility studies. Assuming an expected completion rate of 84%, and aiming to confirm that this proportion exceeds a lower threshold of 75% (a pre-defined feasibility benchmark for planning a future confirmatory trial), we calculated the sample size required to achieve a two-sided 95% confidence interval with a margin of error no greater than 0.09. This yielded a minimum of 64 participants per intervention group. Regarding the control group (standard of care, SOC), we reduced the sample size to N = 32, as the main objective of this pilot study is to evaluate the feasibility of the two intervention arms, not to perform between-group comparisons. We consider this allocation appropriate, as it allows us to explore patterns in secondary outcomes within the SOC group, while preserving statistical efficiency and feasibility in this resource-limited feasibility phase. The unequal randomization ratio (2:2:1) was determined by the investigators and is consistent with the study's primary feasibility objectives.
- We hope this additional rationale adequately addresses the concern, and we have revised the relevant section of the manuscript to clarify these points and can be found on p p. 11-12, lines 405-420: "This feasibility study will explore the rate of completing at least one online module before surgery (outcome criterion 2). This will provide valuable information on what can be expected in a future phase III trial. As one proportion confidence intervals are used to estimate a population proportion, sample size calculation is based on a confidence interval approach. For a future phase III trial, we want to assure that at least 75% of the patients of an iEXPECT group will complete at least one online module before surgery. In our study we expect a completion rate of 84% in each intervention group. Using a two-sided 95% confidence interval (CI) with a lower CI limit of 75%, and an expected completion rate of 84%, we found that each intervention group should have at least 64 patients for the study. As the primary focus lies on assessing the feasibility of the online interventions, the calculated sample size is required for each of the two intervention groups. Since this is a feasibility trial and not designed to detect statistically significant differences between groups, we decided to

allocate only half as many participants (N = 32) to the standard of care (SOC) group. We consider this base size sufficient to provide preliminary insights into secondary outcomes within the SOC group. Accordingly, patients will be randomized in a 2:2:1 ratio across the two intervention arms (iEXPECT enhanced: iEXPECT limited) and the SOC arm, resulting in a total sample size of N = 160. The sample size calculation was performed using PASS14 software (NCSS, LLC, Kaysville, UT, USA; www.ncss.com)."

20. Is December 2025 when the last patient is expected to be recruited, or when the last follow-up occurs?

Response:

- We have added additional information what the intervention consists of on p. 8 lines 179-181: "Recruitment was planned to start in August 2024, and the anticipated completion of recruitment (i.e., when the last patient is expected to be enrolled in the trial) is at the end of December 2025. The first patient was successfully recruited on November 8, 2024."

21. There appears to be a word missing in the last bullet point in the Inclusion Criteria – 5 'hours' or 5 'days' or 5 'months'?

Response:

- The missing word is "days". The change can be seen on p. 6, line 186.

22. When do the patients get approach for recruitment – how long before their surgery does this occur, and how long do they have to consider whether they will consent? Does the anonymous screening data get stored in the study database for both eligible and ineligible patients? Do ineligible patients receive any information about expectation setting? Who assesses the presence of relevant mental disorders as part of the screening process? If new mental disorders are identified, are there referral pathways to ensure appropriate care is provided for the patient?

Response:

- The medical team at the Departments of Cardiac and Thoracic Vascular Surgery informs patients about the study as soon as their surgery is scheduled and they are confirmed to meet the key inclusion criteria—ideally between 21 and 5 days before their surgery. Depending on study center, patients are contacted by phone or receive a flyer with information about the study. We added on p. 6 line 197-203: "The medical team at the Departments of Cardiac and Thoracic Vascular Surgery informs patients about the study as soon as their surgery is scheduled and they are confirmed to meet the key inclusion criteria—ideally between 21 and 5 days before their surgery. In addition to a brief phone call providing initial information, patients receive a brochure enclosed with their surgery confirmation letter. The brochure outlines the study's objectives, procedures, duration, and includes a link and QR code to the study website. On the website, patients complete anonymous screening questions to determine eligibility (e.g., type of surgery, timing, and whether it is their first heart surgery)."
- After patients indicate interest and provide their contact details via the informed consent page, which outlines comprehensive information about the study and participation conditions, they can give preliminary consent by selecting the appropriate checkbox (see p. 6, lines 213). We amended the sentence on p. 6, lines 213-217: "Once initial consent is given,

a study therapist contacts them by telephone to conduct a more detailed screening; during this call, patients are again informed about the study's potential risks, have the opportunity to ask additional questions before giving full informed consent and being formally enrolled. During the phone call, the voluntary nature of participation and the option to withdraw at any time are emphasized."

- All screening data—whether patients are eligible or not—is securely stored in the study database. We added on p. 6 lines 206-208: "All screening data—regardless of eligibility—is securely stored in the study database, with personal identifiers removed or coded in accordance with clinical data confidentiality standards."
- Ineligible patients do not receive any study-specific materials or guidance; they simply continue with standard of care (SOC). We amended in the text on p. 6, lines 204-206: "Ineligible patients receive a message informing them they do not qualify for the study and continue to receive only the SOC without being provided any additional study-specific materials or guidance."
- Screening for mental health conditions is conducted by study therapists, who are psychology students with a bachelor's degree and working toward their master's. If they identify a relevant mental disorder, this is always communicated to the study team, which includes a licensed psychotherapist. Patients are then made aware of any suspected diagnoses, but the study does not guarantee faster access to psychotherapy. Instead, we provide general information about where to seek help; no formal referral pathway is offered. In cases of urgent findings—such as a critical PHQ-9 score—the patient is contacted directly, if possible, in collaboration with the clinic, to ensure immediate support. We emphasize that our goal is to evaluate whether the intervention is helpful, and participation in the study does not confer any additional psychotherapy benefit—patients will need to seek standard routes for scheduling an initial psychotherapeutic consultation themselves, either via telephone or through an online platform, to access mental health care. We amended the text on p. 6-7 lines 221-227: "If patients consent, the presence of relevant mental disorders is assessed using the International Diagnosis Checklists for ICD-10 and the Structured Clinical Interview for DSM-IV (SCID-I) [21] and any incidental findings are communicated to the study team. Patients may choose to be informed of suspected diagnoses, although the study does not expedite psychotherapy access—instead, general guidance on seeking care is provided, without a formal referral pathway. In urgent cases (e.g., critical PHQ-9 scores), patients are contacted directly, if possible, in collaboration with the clinic, to ensure immediate support."

23. What does 'PDI' refer to in the first sentence of 'Randomization'? Please spell out the abbreviation in full and reference it if appropriate. Who created the randomisation schedule and what is their relationship to the study? Are there back-up envelopes in case of technology failure? Are the participants instructed to conceal their randomization from their treating medical team?

Response:

- PDI refers to Pain disability Index. It is a self-report instrument that has been used to assess the degree to which chronic pain interferes with various daily activities. An adapted version (tailored to patients undergoing heart surgery) developed by Tait et al. (1990) will be used. (Tait, R. C., Chibnall, J. T., & Krause, S. (1990). The Pain Disability Index: Psychometric properties. *Pain*, *40*(2), 171–182.)
- A biometrician of the KKS (Christine Heinz) created a randomization script using the software RStudio 2022.12.0. The data management department of the KKS used this code for creating the final randomization list. We added to the text on p. 9, lines 245-247: "A biometrician

from the KKS developed the randomization script using RStudio 2022.12.0, and the KKS Data Management Department utilized this code to generate the final randomization list. It is not planned to use back-up envelopes."

- Participants are not explicitly instructed to conceal their allocation from their treating medical team. However, because the study therapists do not disclose group assignments to medical staff and the online intervention is accessed privately by participants, the treating team remains blind to randomization status by default, minimizing any risk of inadvertent unblinding. We added to the text on p. 7 lines 243-246: "Participants are not explicitly instructed to conceal their group allocation from the treating medical team. However, the medical staff will not receive any information about the group a patient is allocated to and therefore be blind regarding group assignments."

24. The pre-surgical assessments take place with quite a wide range (5-21 days before surgery); how will this wide window be handled during analysis?

Response:

- We don't expect that time from baseline assessment to surgery will impact primary outcome criteria 1 and 3 (number of recruited patients, complete data at 6-month follow-up assessments, acceptance of online modules). For checking if time to surgery is relevant for outcome criterion 2 (completion of at least one online module before surgery) a sensitivity analysis might be conducted.

25. Given the primary outcomes relate to feasibility and acceptability, do participants need to consent to blood sampling, or is that optional?

Response:

- As part of routine clinical care, patients are required to provide blood samples prior to surgery. To date, we have not encountered reluctance from participants to provide additional blood samples for research purposes within the context of the study. Nevertheless, participation in the blood sampling component is entirely optional, and patients may still take part in the study even if they choose not to provide these samples. In the text we added on p. 6, lines 218-221: "As part of routine clinical care, patients are required to provide blood samples prior to surgery; participation in the optional research blood sampling component is voluntary, and patients may still enroll in the study even if they decline to provide study-specific specimens."

26. How long will the assessments take at each timepoint for the participant to complete? Will all assessment questionnaires be undertaken online, or a mixture of paper and online? What retention strategies are in place to encourage participants to complete all timepoints and all assessments at each timepoint? Reminder emails and calls.

Response:

- Participants will require approximately 30 minutes to complete assessments at each timepoint. While questionnaires are primarily administered online via a secure web-based survey platform called UniPark, a paper-based alternative is available through clinical staff in cases of technical difficulties. To promote retention, the study employs personalized email invitations, reminder emails and phone calls, and ensures access to dedicated study staff for support. We added in the text on p. 10, lines 327-333: "At each assessment point (T0–T3),

completion of the psychological questionnaires will require approximately 30 minutes. Participants are encouraged to complete them online via UniPark, although paper-based versions may be provided by clinical staff in cases of technical difficulties, and retention is supported through personalized email invitations, reminder emails and phone calls. These strategies have been shown to enhance compliance and reduce attrition [45], helping to ensure complete data collection across all timepoints."

27. Clarity of outcome measures for a clinical trial is extremely important. Presently, it is hard to identify all the outcome measures as they are dispersed between two sections and intermixed with data analysis approaches. Please amend the two outcome sections to purely describe the outcomes, identifying which are primary and which are secondary and including a definition for each.

Response:

- We thank you for pointing this out. We have amended the outcome sections to clearly distinguish between primary and secondary outcomes, providing a brief definition for each to ensure clarity and alignment with study objectives on p. 9, lines 281-299: "The primary outcomes of the iHEART trial are focused on evaluating the feasibility and acceptance of the iEXPECT intervention and the study design.
  Feasibility
  Feasibility is defined across two dimensions, namely (1) feasibility of the study design, referring to the successful recruitment and retention of participants across multiple study sites (outcome criterion 1), and (2) feasibility of intervention implementation, referring to the patients' engagement with the iEXPECT modules prior to surgery (outcome criterion 2). These outcomes reflect whether the trial can be operationalized as intended and whether participants are willing and able to engage with the digital intervention components.
  Acceptance
  Acceptance is definded as participants' subjective evaluation of the iEXPECT intervention (outcome criterion 3). This includes perceived enjoyableness, usefulness, and the intervention's relevance to their daily social functioning. Ratings are gathered through self-report at the end of each module and via a standardized satisfaction questionnaire (CSQ-I) [27] after intervention completion (T1) and at 6-month follow-up (T3). Together, these outcomes will inform the practicality and perceived value of the intervention from the patient's perspective and provide critical insight for the planning of a future full-scale efficacy trial."
- And on p. 9, line 302-320: "Secondary outcomes include multimodal assessments (i.e., self-ratings, expert ratings, clinical facts, biological measures) that were selected to capture a comprehensive picture of psychological, behavioral, and clinical processes that may be affected by the intervention and are relevant for evaluating its broader impact and future phase III trial feasibility. These include measures of preoperative anxiety and need for information (APAIS) [28, 29], general anxiety and depression (PHQ9 and GAD-7) [30, 31], and treatment expectations (TEX-Q) [32], all of which are known to influence surgical outcomes and psychological readiness [33]. We also included constructs such as attitudes toward internet-based interventions (APOI) [34], subjective illness beliefs (B-IPQ) [35], social support (ESSI-D)([36], and trait optimism (LOT-R) [37], which may moderate treatment engagement and response. Furthermore, indicators of digital health literacy (GR-eHEALS, revised German eHealth literacy scale) [38], physical activity (IPAQ) [39], perceived stress (PSS) [40, 41],

illness-related disability caused by the current medical condition (PDI, adapted version) [22], quality of life (SF-12) [42], and treatment satisfaction (i.e., rating of fulfilment/violation of pre-treatment expectations at the end of the trial) may provide a multidimensional view of patient functioning across time. To explore potential adverse effects and unmet needs, we assess negative side effects (NEQ) [43] and prior pre-treatment experiences. The following clinical parameters are also included to explore associations between psychological factors and physical recovery: rehospitalization; length of stay; length of time at intensive care unit; planned and realized surgery; EURO-Score II [44]; blood pressure; LVEF; NYHA score; complications; inflammatory processes (CRP, IL-6, IL-8); HRV (i.e., triangular index). These outcomes will be analyzed descriptively to identify relevant trends, inform intervention refinement, and guide power calculations for a future phase III trial."

28. Following, please include a paragraph on data analysis that includes details on the descriptive statistics that will be presented and what outcomes they will be presented for, any regression modelling that will be used and if so, what covariates will be included and what effect estimates and associated measure of variability will be presented, any subgroup or sensitivity analyses, howe missing data will be handled, how the temporal nature of the data will be incorporated in analyses, and how the type I error will be incorporated.

Response:

- As this is a feasibility study, statistical analyses will be conducted primarily using descriptive methods. The results will serve to evaluate the feasibility and acceptance of the online interventions and to inform sample size calculations for a future phase III trial. While the study is not powered to detect efficacy, exploratory correlation and regression analyses may be performed to examine relationships among secondary outcome variables and to generate hypotheses for subsequent confirmatory research. We added in the manuscript on p. 11, lines 376-402: "The hypotheses of primary interest refer to the feasibility and acceptability of the iEXPECT intervention as an internet-based intervention, which will be assessed based on three criteria:
    - Feasibility of the study design is assumed if at least one participant per week per center can be recruited (approximately 160 participants from three centers over 53 weeks), and >49% of all randomized patients provide complete data for the 6-month follow-up assessments, including biomarkers. This criterion is checked collectively for all groups and individually for each group. Absolute and relative frequencies with two-sided 95% confidence intervals will be reported.
    - Feasibility of the internet-based intervention is assumed if >75% of the randomized patients in each iEXPECT arm complete at least 1 (>0) online module before the surgery. The completion rate will be assessed separately for each intervention group. Two-sided 95% confidence intervals will be given.
    - Acceptance will be assessed by self-report ratings at the end of each online module and with the CSQ-I after the intervention (T1) and the 6 months follow-up (T3). To evaluate a session's/ the intervention's enjoyableness, usefulness, and effect on daily social functioning using a 5-point scale (1 = fully disagree, 2 = disagree, 3 = not sure, 4 = agree, 5 = fully agree; examples for items: 'This session/intervention was useful and sensible', 'I would recommend this session/intervention to others', 'This session/intervention lived up to my expectations'). Acceptance of the internet-based intervention is assumed if the mean rating for overall acceptance/satisfaction with

the intervention (a mean score will be calculated for the assessments after completion of each of the 4 modules) is positive (>3.4 (better than 'not sure') on a 5-point Likert scale from 1 to 5). Each intervention group will be analyzed separately. Secondary outcomes will be analyzed descriptively to identify relevant trends, inform intervention refinement, and guide power calculations for a future phase III trial. While the study is not powered to detect efficacy, exploratory correlation and regression analyses may be performed to examine relationships among secondary outcome variables and to generate hypotheses for subsequent confirmatory research."

29. Please include a section on data monitoring – how will source data verification be undertaken. How will participant identifiability be guaranteed?

Response:

- We included a section on data monitoring which be found on p. 12 lines 423-433: "To ensure confidentiality, all study data will be de-identified using a unique coding system (e.g., first patient in Marburg "176-001"), with personal identifiers replaced by anonymized codes. Only study investigators will have access to the coded dataset. Results will be published exclusively in anonymized form. All anonymous screening data—regardless of eligibility—is securely stored in the study database, with personal identifiers removed or coded in accordance with clinical data confidentiality standards. As part of quality assurance, a random sample of entries will undergo source document verification; for example, surgical dates in REDCap will be cross-checked against patient medical records to confirm accuracy. The study management team maintains a subject identification list within REDCap, enabling targeted verification in collaboration with clinical sites. Access to the data is strictly limited to investigators, and all output will comply with the CONSORT 2025 Statement guidelines. Data analysis and manuscript preparation will be carried out by study coordinators, with subsequent submission to peer-reviewed journals and presentation at international conferences. Participants may request study results following their involvement."

30. An IDMC of only two people seems small; please defend the size of this committee, and the lack of inclusion of clinical trialists/statisticians/consumers.

Response:

- In the PSYHEART-II trial, the Independent Data Monitoring Committee (IDMC) consisted of three members. Given that the present study is a feasibility trial with a smaller sample size and no dedicated funding was secured for the IDMC, available resources are limited. Given these circumstances and following the suggestions of the KKS Marburg (the methological experts for conducting clinical trials), the composition of the IDMC was streamlined and two experts were considered sufficient. An expansion of the IDMC is planned for the subsequent efficacy trial. The IDMC members have relevant expertise to ensure participant safety and data integrity within the constraints of this preliminary phase. We have included a brief explanation on p. 12, lines 442-447: "As this study is a feasibility trial with a smaller sample size and no dedicated funding for an IDMC, two members were deemed sufficient for the committee, compared to three in the larger PSY-HEART-II trial [56]. While the inclusion of clinical trialists, statisticians, and consumer representatives is planned for the subsequent full-scale study, the current IDMC ensures appropriate methodological and clinical oversight within the scope and resource constraints of this preliminary phase."

31. What reporting guideline will be used when reporting the study results?

- The study results will be reported in accordance with the CONSORT 2025 Statement to ensure transparent and standardized reporting of trial methodology and findings. We added on p. 12, lines 429-431: "While the study is ongoing, access to the data is strictly limited to the investigators, and all output will comply with the CONSORT 2025 Statement guidelines."

ETHICS AND DISSEMINATION

32. Will participants be interviewed? It's unclear when and how this is occurring. If they are, please ensure this is clear in the Methods, including who is interviewing them, how the interview is recorded, how the transcript is generated, where the audio/video is stored, etc.

Response:

- We acknowledge the potential for confusion in the original formulation and have revised the section accordingly to improve clarity on p. 13, lines 473-474: "Patients who decline participation at specific assessment time points will still be contacted at later time points, where appropriate, to explore reasons for withdrawal and gather relevant follow-up information."

33. Please separate Ethics and Dissemination sections and place Ethics higher up in the Methods.

Response:

- We have now separated the Ethics and Dissemination sections and placed them before the discussion (p. 13, lines 460-484).

34. The Dissemination approach is very limited; please consider novel and consumer-informed methods of dissemination.

Response:

- We added on p. 13, lines 481-484: "Study findings will be disseminated to participants, the public, policymakers, and the scientific community via peer-reviewed publications, and conference presentations. Furthermore, we aim to compose a text that is comprehensible to laypersons and make it available to the German Heart Foundation for potential publication on their website, thereby reaching the public."

FIGURE

35. It cannot be known how many patients will be screened and how many will end up in each group; I would suggest removing any numbers from the figure.

Response:

- Done.

VERSION 2 - REVIEW

Reviewer **2**

Name **Gibbons, Kristen**

Affiliation **The University of Queensland, Child Health Research Centre**

Date **05-Aug-2025**

COI

Thank you for your attention to the previous comments. There are only a few minor outstanding queries:

1. When discussing dissemination approaches in the abstract, "potential support from the German Heart Foundation" does not sound like a secured dissemination approach. If this pathway to dissemination has not been secured, suggest being a little broader, e.g. referring to "relevant consumer advocacy groups".

2. How were the thresholds for the outcome measures (e.g. >=1 participant per week per centre, >=49% randomized patients) chosen?

3. With respect to the sample size, why was an expected completion rate of 84% chosen? i.e. where was the 84% derived from?

4. Please specify that the biometrician from KKS who developed the randomisation schedule has no involvement with the trial analysis or data collection (if this is the case).

5. The response to question 24 states a sensitivity analysis may be undertaken, however this is not outlined in the analysis plan; please amend.

6. The response to question 29 states that study coordinators will undertake the data analysis and manuscript preparation; it would be usual practice for statisticians and investigators to undertake this component of work, just be aware this may be questioned.

7. It is recommended to be explicit that no p-values will be presented as the study is not powered to do so. What statistic will the confidence intervals be presenting - will it be the difference between two groups (and if so, which groups), or a point estimate per group? Please also outline in the data analysis plan how descriptive statistics of the study groups will be analysed and reported. Please defend the threshold of 3.4 for a positive acceptance.

VERSION 2 - AUTHOR RESPONSE

Reviewer #2 Dr. Kristen Gibbons, The University of Queensland

Comments to the Author: Thank you for your attention to the previous comments. There are only a few minor outstanding queries:

Response:

- We thank you for the positive feedback and the constructive comments, which have further improved the quality of the manuscript.

1. When discussing dissemination approaches in the abstract, "potential support from the German Heart Foundation" does not sound like a secured dissemination approach. If this pathway to dissemination has not been secured, suggest being a little broader, e.g. referring to "relevant consumer advocacy groups".

Response:

- Thank you for pointing this out. Indeed, we did not secure a pathway to dissemination with the German Heart Foundation. Therefore, we amended on p. 3 l. 80-81: "Results will be shared via publications, conferences, and public outreach with relevant consumer advocacy groups." And on p. 13, l. 507 – 508: "Furthermore, we aim to compose a text that is comprehensible to laypersons and make it available to relevant consumer advocacy groups (e.g., the German Heart Foundation) for potential publication on their website, thereby reaching the public."

2. How were the thresholds for the outcome measures (e.g. >=1 participant per week per centre, >=49% randomized patients) chosen?

Response:

- The threshold of ≥1 participant per week per centre was chosen based on feasibility considerations. Specifically, it was derived from the assumption that a Phase III trial involving *X* centres over a recruitment period of *Y* would be achievable if each site enrolled approximately one participant per week. This benchmark was therefore intended to ensure that the projected recruitment targets could realistically be met within the planned timeline. Similarly, the ≥49% randomized patients threshold was selected to reflect a level of screening-to-randomization efficiency that would be considered acceptable for maintaining trial feasibility, while also accounting for typical attrition rates observed in comparable studies.
- We amended the text on p. 11, l. 381-385: "The recruitment threshold of ≥1 participant per centre per week was based on the assumption that a Phase III trial with *X* centres could achieve its enrolment target within a recruitment period of *Y* under this rate. The ≥49% randomization threshold was chosen to reflect an acceptable screening-to-randomization efficiency while accounting for expected attrition."

3. With respect to the sample size, why was an expected completion rate of 84% chosen? i.e. where was the 84% derived from?

Response:

- This feasibility study assesses the proportion of patients completing at least one online module before surgery (feasibility criterion 2). For a subsequent phase III trial, our aim is that at least 75% of patients in the iEXPECT group complete one module; therefore, 75% was set as the required lower bound of the 95% confidence interval. We chose 84% as the expected completion rate because it provides a realistic buffer above this threshold while keeping the sample size feasible. Based on KKS calculations (see table below), assuming 83% would require 85 patients per arm (a 33% increase with little additional benefit), whereas assuming 85% would reduce the sample to 49 per arm but risk underpowering the study if the true rate were slightly lower. Thus, 84% was selected as a balanced assumption that ensures the feasibility criterion can be demonstrated robustly in this study and increases the likelihood of achieving it in a subsequent confirmatory trial. For more clarity we added on p. 12, l.421 – 423: "In our study we expect a completion rate of 84% in each intervention group, because it provides a meaningful buffer above the 75% feasibility threshold and ensures

that the lower bound of the 95% confidence interval will remain above this criterion, while keeping the required sample size feasible."

True Feasibility	Sample size
0.76	7007
0.77	1701
0.78	732
0.79	398
0.80	246
0.81	164
0.82	116
0.83	85
0.84	64
0.85	49

4. Please specify that the biometrician from KKS who developed the randomisation schedule has no involvement with the trial analysis or data collection (if this is the case).

Response:

- Excellent point. We amended the text for more clarity on p. 7, l. 237-240: "A biometrician from the KKS, who had no involvement in trial analysis or data collection developed the randomization script using RStudio 2022.12.0, and the KKS Data Management Department utilized this code to generate the final randomization list."

5. The response to question 24 states a sensitivity analysis may be undertaken, however this is not outlined in the analysis plan; please amend.

Response:

- Thank you for pointing this out. We amended the section in the manuscript on p. 11, l. 388-393: "Feasibility of the internet-based intervention is assumed if >75% of the randomized patients in each iEXPECT arm complete at least 1 (>0) online module before the surgery. The completion rate will be reported as a point estimate per intervention group, and two-sided 95% confidence intervals will be provided. To assess whether time to surgery influences outcome criterion 2 (completion of at least one online module before surgery), a sensitivity analysis will be considered."

6. The response to question 29 states that study coordinators will undertake the data analysis and manuscript preparation; it would be usual practice for statisticians and investigators to undertake this component of work, just be aware this may be questioned.

Response:

- We acknowledge that our wording was imprecise in the initial response. Data analysis and manuscript preparation will be conducted jointly by the study statisticians and investigators. We amended the section on p. 12, l. 444-446: "Data analysis and manuscript preparation will

be carried out jointly by the study statisticians and investigators, followed by submission to peer-reviewed journals and presentation at international conferences."

7. It is recommended to be explicit that no p-values will be presented as the study is not powered to do so. What statistic will the confidence intervals be presenting - will it be the difference between two groups (and if so, which groups), or a point estimate per group? Please also outline in the data analysis plan how descriptive statistics of the study groups will be analysed and reported. Please defend the threshold of 3.4 for a positive acceptance.

Response:

- The confidence intervals will present point estimates per group, not differences between groups. Specifically, primary outcome 1: The proportion of patients with complete data for the 6-month follow-up assessments (including biomarkers) will be reported as a point estimate per group. Two-sided 95% confidence intervals will be given.
- For primary outcome 2: The proportion of patients who complete at least 1 online module before the surgery will be reported as a point estimate per intervention group. Two-sided 95% confidence intervals will be given.
- Primary outcome 3: Mean rating of acceptance/satisfaction with the intervention will be presented for each online module. In particular, an overall mean rating across the four modules/predefined timepoints of assessment will be calculated. We amended in the text on p. 11, l. 378 - 407: "Feasibility of the study design is assumed if at least one participant per week per center can be recruited (approximately 160 participants from three centers over 53 weeks), and >49% of all randomized patients provide complete data for the 6-month follow-up assessments, including biomarkers. The recruitment threshold of ≥1 participant per centre per week was based on the assumption that a Phase III trial with X centres could achieve its enrolment target within a recruitment period of Y under this rate. The ≥49% randomization threshold was chosen to reflect an acceptable screening-to-randomization efficiency while accounting for expected attrition. The proportion of patients with complete data for the 6-month follow-up assessments (including biomarkers) will be reported as a point estimate per group. Two-sided 95% confidence intervals will be given.
- Feasibility of the internet-based intervention is assumed if >75% of the randomized patients in each iEXPECT arm complete at least 1 (>0) online module before the surgery. The completion rate will be reported as a point estimate per intervention group, and two-sided 95% confidence intervals will be provided. To assess whether time to surgery influences outcome criterion 2 (completion of at least one online module before surgery), a sensitivity analysis will be considered.
- Acceptance will be assessed by self-report ratings at the end of each online module and with the CSQ-I after the intervention (T1) and the 6 months follow-up (T3). To evaluate a session's/the intervention's enjoyableness, usefulness, and effect on daily social functioning using a 5-point scale (1 = fully disagree, 2 = disagree, 3 = not sure, 4 = agree, 5 = fully agree; examples for items: 'This session/intervention was useful and sensible', 'I would recommend this session/intervention to others', 'This session/intervention lived up to my expectations'). Acceptance of the internet-based intervention is assumed if the mean rating for overall acceptance/satisfaction with the intervention (calculated after completion of each of the four modules) exceeds 3.4. This threshold was chosen to reflect a tendency toward agreement

rather than neutrality (>3 = “not sure”) and is supported by prior data [12] suggesting an expected mean of 3.7 (SEM = 0.14), making >3.4 a conservative and statistically robust criterion. Each intervention group will be analyzed separately and mean rating of acceptance/satisfaction with the intervention will be presented for each online module. An overall mean rating across the four modules/predefined timepoints of assessment will be calculated.”

- Baseline characteristics will be presented by group. For categorical variables, number of patients per category will be reported as well as the category proportion. Number of missing values will also be presented. For continuous variables, mean and standard deviation will be reported. Primary and secondary outcome variables will be analyzed in the same way at the predefined timepoints. We added on p.11, l. 409 – 412: “Baseline characteristics will be presented by group. For categorical variables, number of patients per category will be reported as well as the category proportion. Number of missing values will also be presented. For continuous variables, mean and standard deviation will be reported. Primary and secondary outcome variables will be analyzed in the same way at the predefined timepoints.”
- The threshold of 3.4 for defining positive acceptance was based on two complementary considerations. First, because the 5-point Likert scale ranges from 1 = fully disagree to 5 = fully agree, we wanted to ensure that the average rating reflected a tendency toward agreement rather than neutrality; thus, the threshold was set above “not sure” (3) and closer to “agree” (4). Second, we assumed the following response distribution based on a previous PSY-HEART-I study study (DOI: 10.1159/000485430) on the 5-point Likert scale: 1 = 5%, 2 = 10%, 3 = 15%, 4 = 50%, 5 = 20%. This distribution corresponds to a mean of 3.7 with a standard error of 0.14. Considering the lower bound of the mean minus one standard error (3.56), a conservative cut-off of >3.4 was selected. This ensures that positive acceptance is defined in a way that is both statistically robust and consistent with observed response patterns in comparable studies.
- For more clarity we amended the paragraph on p.11, l 400 – 404: “Acceptance of the internet-based intervention is assumed if the mean rating for overall acceptance/satisfaction with the intervention (calculated after completion of each of the four modules) exceeds 3.4. This threshold was chosen to reflect a tendency toward agreement rather than neutrality (>3 = “not sure”) and is supported by prior data [12] suggesting an expected mean of 3.7 (SEM = 0.14), making >3.4 a conservative and statistically robust criterion.”
